# Nanocomposites as biomolecules delivery agents in nanomedicine

**DOI:** 10.1186/s12951-019-0479-x

**Published:** 2019-04-03

**Authors:** Magdalena Bamburowicz-Klimkowska, Magdalena Poplawska, Ireneusz P. Grudzinski

**Affiliations:** 1 Department of Applied Toxicology, Faculty of Pharmacy, Medical University of Warsaw, Banacha 1 Str, 02-097 Warsaw, Poland; 2 Department of Organic Chemistry, Faculty of Chemistry, Warsaw University of Technology, Noakowskiego 3 Str, 00-664 Warsaw, Poland

## Abstract

Nanoparticles (NPs) are atomic clusters of crystalline or amorphous structure that possess unique physical and chemical properties associated with a size range of between 1 and 100 nm. Their nano-sized dimensions, which are in the same range as those of vital biomolecules, such as antibodies, membrane receptors, nucleic acids, and proteins, allow them to interact with different structures within living organisms. Because of these features, numerous nanoparticles are used in medicine as delivery agents for biomolecules. However, off-target drug delivery can cause serious side effects to normal tissues and organs. Considering this issue, it is essential to develop bioengineering strategies to significantly reduce systemic toxicity and improve therapeutic effect. In contrast to passive delivery, nanosystems enable to obtain enhanced therapeutic efficacy, decrease the possibility of drug resistance, and reduce side effects of “conventional” therapy in cancers. The present review provides an overview of the most recent (mostly last 3 years) achievements related to different biomolecules used to enable targeting capabilities of highly diverse nanoparticles. These include monoclonal antibodies, receptor-specific peptides or proteins, deoxyribonucleic acids, ribonucleic acids, [DNA/RNA] aptamers, and small molecules such as folates, and even vitamins or carbohydrates.

## Introduction

Nanoparticles (NPs) are atomic clusters of crystalline or amorphous structure that possess unique physical and chemical properties associated with a size range of between 1 and 100 nm. The nano-sized dimensions of NPs are similar to the size of many vital biomolecules such as antibodies, membrane receptors, nucleic acids, and proteins. These mimicking size features, together with their high surface area to volume ratio, make nanoparticles a powerful tool in modern nanomedicine. The increasing demand of clinical applications and the development of nanobiotechnology have substantially promoted bioengineering strategies for a variety of nanosystems in different areas of applications such as molecular imaging, point-of-care diagnostics, and targeted therapies [1–8]. It seems reasonable to note that the extraordinary properties of nanoparticles have led to an exponential increase in the reactivity at both cellular and molecular levels. To meet these challenges, the design of multi-functional nanoparticles could significantly improve already existing knowledge. Monofunctional nanoparticles have a single feature. For example, in cells and tissues, a nanoliposome can transport drugs but does not have the ability to distinguish between healthy and unhealthy cells or tissues [9]. In contrast, multifunctional nanoparticles combine different functionalities in a single property-designed nanocomposite. For example, a nanoparticle could be functionalized with an appropriate moiety possessing a specific targeting function that recognizes the unique surface signature of its target cells [10, 11]. Simultaneously, the same nanoparticle can be modified with an imaging agent to monitor the transport process and evaluate some pathological features. Last but not the least, the nanoparticle can be linked to a moiety to evaluate the therapeutic efficacy of a drug and to reduce its side effects. Such multifunctional nanoparticles will be defined here as theranostics (combination of therapeutics and diagnostics). As these NPs come into contact with cells and body fluids, they are exposed to many self-assembled changes in terms of ionic strength, pH, protein concentrations, and compositions, all of which affect the properties of NPs and their interactions with the cells [7, 12]. The unique behavior of NPs in terms of cellular endocytosis, transcytosis, neuronal and circulatory translocation and distribution that make them desirable for medical therapeutic or diagnostic applications, may also be associated with potential toxicity [13, 14]. Efforts have been made to optimize the methodology applied in the area of nanotoxicology, especially theoretical modeling, to keep up with the pace with which novel NPs are being developed due to safety by design approaches [15, 16].

Rapid technological progress has led to the use of a number of advanced nanomaterials for constructing smart multifunctional nanosystems for targeted diagnosis and therapies of various diseases including cancers [6, 16–18]. In personalized therapies, the treatment approach should ideally and exclusively target the anomalous cancerous cells and tissue with no or minimal impact on the normal cells. The present review provides an overview of the most recent (mostly last 3 years) scientific achievements related to different biomolecules used to enable targeting capabilities of highly diverse nanoparticles. These include monoclonal antibodies, receptor-specific peptides or proteins, deoxyribonucleic acids, ribonucleic acids, aptamers, and small molecules as folates and even vitamins or carbohydrates.

## Protein delivery

Delivery of proteins or peptides using NPs is still in its infancy in cancer nanotechnology. Encapsulation of proteins in nanoparticles can greatly improve the properties of proteins, such as their stability against denaturation and degradation processes due to proteases and enable to use natural proteins isolated from their intrinsic localization in living organisms for biomedical applications. Numerous excellent reviews focusing on silica NPs for therapeutic and other applications can be found elsewhere [19, 20].

### Graphene oxide

**Fig. 1:**
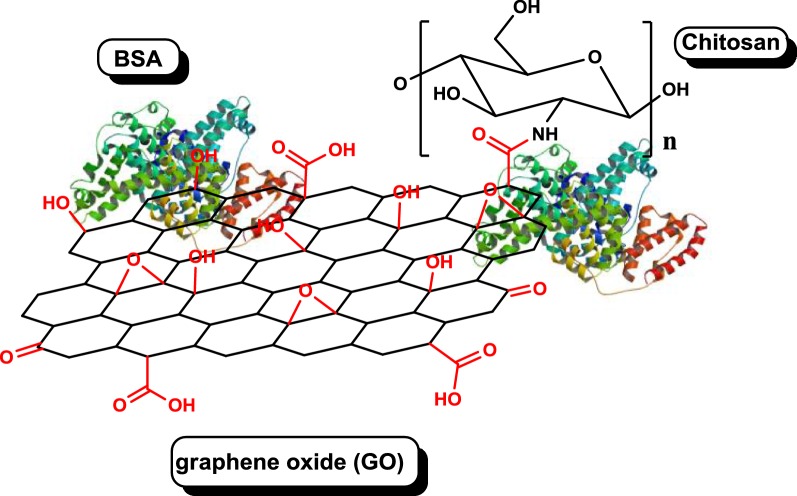
Schematic diagram of GO functionalized with chitosan [22]

Most studies to date have used chitosan-, polyethylene glycol (PEG)- or poly(ethylene glycol) dimethacrylate-functionalized graphene oxide (GO) and reduced graphene oxide (rGO) for protein or peptide delivery. Only a few studies have taken advantage of the ability of graphene to form multilayer systems by using layer-by-layer technology synthesis [9, 21]. Bovine serum albumin (BSA) as a model protein has often been used to prove the possibility of effective adsorption of proteins and peptides onto NPs and their enhanced stability against proteolytic enzymes [22, 23]. Moreover, few studies have attempted to load therapeutic peptides and proteins on GO and demonstrate their functional activity following delivery. Graphene-based nanoparticles have shown a great potential as nanoscale carriers for protein delivery. Efforts have been devoted to explore the potential peptide and protein carriers for anticancer agents including doxorubicin (DOX), cisplatin (Pt(IV)), and others. Emadi et al. functionalized GO with chitosan to produce a new nanocarrier for proteins to protect them against proteolysis, extend their half-life, and retain their activity and stability in biological environments. Figure 1 shows the schematic diagram of GO functionalized with chitosan [22].

Bovine serum albumin was loaded on the native and chitosan-functionalized GO to investigate the loading efficacy and stability against trypsin. The loading efficacy of BSA increased from 26 to 63% when the native GO or chitosan-modified GO was applied. There was no significant change in the intact protein in the GO-BSA and chitosan-functionalized GO-BSA solutions after 30 min and 1 h of exposure to trypsin. However, free BSA was completely digested after 1 h. GO and chitosan-modified GO protected the protein against enzymatic cleavage. Chitosan-decorated GO nanocarrier showed higher efficacy of BSA loading and was more biocompatible than nonfunctionalized GO. Because of its unique properties, functionalized graphene nanostructure could protect proteins against enzymolysis [22]. In other recent studies, Kurapati and Raichur used layer-by-layer assembly technique to prepare GO/poly(allylamine hydrochloride) multilayer capsules. These hybrid capsules showed special ‘‘core–shell’’ loading property. This material also had unique permeability characteristic, which allowed the adsorption of proteins and drugs on GO sheets in shells of the capsules. Poly(allylamine)-modified nanosystem was characterized by high adsorption capability for proteins through simple non-covalent interactions such as hydrogen bonding between oxygen functional groups of GO and nitrogen/oxygen functional groups of BSA and electrostatic interactions along with strong hydrophobic and π–π stacking forces [24].

Graphene oxide multilayer systems have been reported by Hong et al. as a platform technology for the release of proteins in preprogrammed sequences over long time periods. Layer-by-layer assembly, which involves the alternating adsorption of multivalent charged molecular species to build a film, is an alternative approach to traditional polymer-based delivery systems. Using ovalbumin as a model protein, the authors of this study prepared a layer-by-layer assembly using GO and cationic poly(β-amino ester). As the number of GO layer increased, the time required to release ovalbumin from the GO multilayer assembly also increased. Human HSCs in vitro model were used in release studies. For example, a 5-layer GO and poly(β-amino ester) assembly required 32 days to release 75% of bound ovalbumin [21].

Multiple anticancer agents designed as a single nanocarrier can optimize their pharmacokinetic profiles and biodistribution behaviors in a spatiotemporal co-delivery mode, resulting in a more efficient synergistic antitumor activity, compared with the conventional drug mixtures [25]. Jiang et al. developed a novel polyethylene glycol (PEG)-functionalized GO nanostructure as a new cellular protease-mediated co-delivery system that integrate membrane-related proteins and intracellular-functioning small-molecule drugs. Such GO nanocarrier was enabled to efficiently release its cargo, namely tumor necrosis factor (TNF)-related apoptosis-inducing ligand and doxorubicin (DOX), in a site-specific manner. Taking advantage of furin presence on the cell membrane, the furin-cleavable peptide was inserted between PEG and tumor necrosis factor-related apoptosis-inducing ligand to start the release of TNF-related apoptosis-inducing ligand in the tumor environment. The GO nanocarrier with the possibility of co-delivery of furin-cleavable TRAIL and doxorubicin application was shown to inhibit tumor growth in A549-xenografted mice more effectively than co-delivery of furin-noncleavable tumor necrosis factor-related apoptosis-inducing ligand and doxorubicin on GO, and delivery of tumor necrosis factor-related apoptosis-inducing ligand and doxorubicin using GO alone [26]. More recently, Yemisci et al. [27] showed that a large peptide such as basic fibroblast growth factor and a small peptide inhibitor of caspase-3 (z-DEVD-FMK) can be effectively transported to the brain after systemic administration by incorporating these peptides to brain-targeted nanoparticles. NPs were prepared by loading either peptide to chitosan–polyethylene glycol-biotin graft polymers followed by conjugating with antibodies directed against the transferrin receptor-1 on brain endothelia to induce receptor-mediated transcytosis across the blood–brain barrier (BBB). Neuroprotection was not observed when receptor-mediated transcytosis was inhibited with imatinib or when basic fibroblast growth factor-loaded NPs were not conjugated with the targeting antibody, which enables them to cross the BBB. Nanoparticles targeted to the brain are promising drug carriers to transport large and small BBB-impermeable therapeutics for neuroprotection against stroke and different glioma cancers [3, 27–30].

### Other carriers

In recent years, the combination of chemotherapy and light irradiation has been developed as a novel approach to enhance the cytotoxicity of anticancer drugs at lower doses by enhancing cellular drug uptake or regulating drug release. Li and colleagues developed a novel human serum albumin protein-based nanocarrier system, which combines the photoactivatable Pt(IV) antitumor prodrug for achieving the controlled release and a fluorescent light-up probe, for the evaluation of drug action and efficacy. The constructed human serum albumin-decorated Pt(IV) platform could be locally activated by light irradiation to release the active Pt species, which results in augmented cell death for both drug-sensitive A2780 and cisplatin-resistant A2780cis cell lines. Simultaneously, cytotoxicity caused by light-controlled drug release would further lead to cellular apoptosis and trigger the activation of caspases 3, a crucial protease enzyme in the apoptotic process. Such a unique design using maleimide-modified human serum albumin nanoconjugates may present a successful platform for biocompatible protein-based nanocarrier for drug delivery with controlled drug release and strengthened therapeutic effects, and thus allows real-time monitoring of antitumor drug efficacy [31].

## Enzymes

**Fig. 2:**
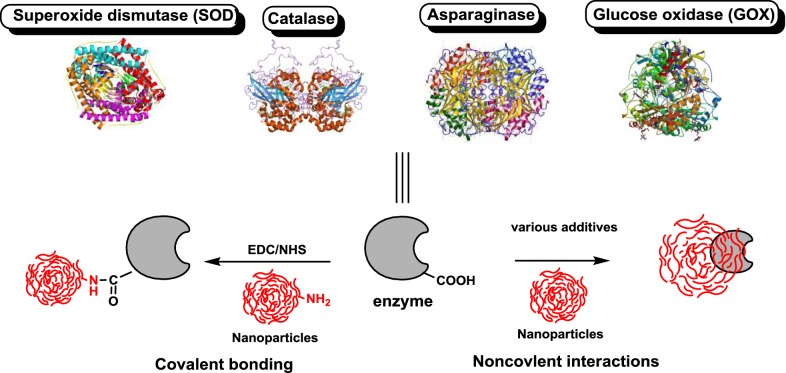
Enzyme immobilization on nanocarriers through covalent binding or physical absorption processes. EDC/NHS is a very common coupling method for creation of amide bonds. This coupling method uses chemical linkers that react with certain groups on the molecules. Therefore, the reaction is more specific and controllable than the passive absorption method, and the number of covalently conjugated ligands can be optimized to the particular application

Different types of nanocarriers have found important potential applications in enzyme immobilization. Enzymes can be linked to nanoparticles by physical absorption or by covalent binding. A general scheme of enzyme combination with nanoparticles is shown in Fig. 2.

Enzymes utilized as therapeutic agents give many advantages over conventional drugs. Enzymes exhibit high specificity and affinity for their target substrates, which can result in a reduction in toxicity and avoiding side effects related to a lack of selective activity. The catalytic features of enzymes allow them to convert target molecules to desired products. This phenomenon could lead to administration of smaller quantities of the therapeutics. Enzyme capabilities make them uniquely potent therapeutics for carrying out activities that conventional drugs do not possess [32]. The large diversity of enzymatic functions and corresponding therapeutic activities has initiated a wide range of research on their possible clinical applications [33].

To avoid disadvantages related to enzyme usage, rapid clearance, and the potential of off-target interaction and toxicity, the encapsulation of enzymes is frequently used. Muzykantov [34] and others described coupling of polyethylene glycol to the antioxidant enzymes, superoxide dismutase (SOD) and catalase, and encapsulating them in liposomes. Such strategies may provide increase in enzyme bioavailability and enhancement of their protective effect [35]. Because reactive oxygen species (ROS) are important factors in many clinical diseases, the direct delivery of antioxidant enzymes into the targeted cells is very desirable for therapeutic purposes.

### Superoxide dismutases

Superoxide dismutases (SOD) are enzymes that catalyze the dismutation of superoxide into oxygen and hydrogen peroxide. Among these enzymes, Cu/ZnSOD and MnSOD are more important isoforms. They work to protect cells against toxic products released during aerobic respiration [34]. Known for their anti-inflammatory activity, they are currently of particular interest. To optimize the stability and activity of superoxide dismutase, Falahati et al. immobilized this enzyme by physical adsorption on mesoporous silica nanoparticles comprising an aminopropyl moiety. No changes were observed in the enzyme structure when it was adsorbed on the aminopropyl-functionalized mesoporous silica carriers. Moreover, the activity of the immobilized enzyme was higher that of the free enzyme and did not change with increasing temperature. After immobilization, the enzyme was more stable and retained its functional structure [36].

A novel polymer–enzyme conjugate was prepared through the conjugation of Cu/Zn superoxide dismutase to a macromolecule carrier, *O*-(2-hydroxyl)propyl-3-trimethyl ammonium chitosan chloride, by Xu et al. to improve the therapeutic potential of SOD. The constructed nanosystem increased the catalytic activity and bioavailability of SOD as well as prolonged its half-life. It exhibited low cytotoxicity to cells and superior membrane permeability compared with native SOD. The conjugate, alone or incubated with alpha-amylase, significantly protected cell viability from ionizing radiation-induced oxidative damage by scavenging intracellular ROS. In vivo SOD nanocarrier remarkably attenuated ROS-induced oxidative damage and protected the tissues in mice [37].

It is known that enzymes cannot penetrate the BBB, which necessitates the development of delivery routes to the central nervous system. Reddy and Labhasetwar used poly(lactic-*co*-glycolic acid) (PLGA) nanoparticles as SOD carriers. They demonstrated PLGA efficacy in a rat focal cerebral ischemia–reperfusion injury model after localized brain delivery via the intracarotid route. The suggested mechanism of efficacy of SOD-nanoparticles appears to be due to protective effect of SOD by neutralizing the deleterious effects of ROS formed following ischemia–reperfusion [38]. In his earlier work, Reddy demonstrated better intracellular neuronal uptake of PLGA-encapsulated SOD over the protein in solution. Moreover, the results demonstrated that PLGA-NPs are compatible with human neurons, and the neuroprotective effect of SOD-nanoparticles was observed in a dose-dependent manner [39].

Immobilization of enzymes on nanomaterials allows to create nanovaccines. Cu/ZnSOD and MnSOD are typically localized in eukaryotes’ cytosol in the mitochondrial matrix, while FeSODs have been found in prokaryotes and protozoa. Two closely related FeSODs have been identified in *Leishmania chagasi*, which were localized within the glycosomes in high levels and were found to play an important role in the survival and growth of *Leishmania* within human macrophages. To develop a new nanovaccine for leishmaniasis, recombinant *Leishmania* superoxide dismutase was loaded onto chitosan nanoparticles. This formulation of SOD in biodegradable and stable chitosan nanoparticles can increase the immunogenicity toward cell-mediated immunity (TH1 cells producing IgG2a in BALB/c mice); this approach is effective in *Leishmania* eradication and could be presented as a single-dose nanovaccine for leishmaniasis [40].

### Catalase

The vascular endothelium is a prime target for drug delivery. Muro et al. attempted to transfer catalase into the endothelial cells. They utilized anti-ICAM-1 antibody for target latex microspheres with biotinylated catalase. Intercellular adhesion molecule-1 (ICAM-1) as a plasma membrane protein can upregulate and is functionally involved in inflammation and thrombosis process. Intracellular anti-ICAM/catalase nanoparticles were active and endothelial cells were resistant to H_2_O_2_-induced oxidative injury for 1–2 h after nanoparticle uptake. Moreover, cell culture pretreatment with chloroquine and nocodazole increased the duration of antioxidant protection by decreasing the extent of anti-ICAM/catalase degradation. Importantly, internalization of these anti-CAM nanoparticles is distinct from clathrin- and caveolin-mediated endocytosis. Anti-CAM nanoparticle uptake depends on signaling induced by CAM clustering and represents a unique actin-dependent process that require the activation of protein kinase C, Src kinase, and Rho kinase (CAM-mediated endocytosis) [41]. This characteristics of ICAM targeting and CAM-mediated endocytosis suggests that drug vectors may be designed for preservation in the endocytic compartment with the potential to enhance their therapeutic effects.

### Glutathione peroxidase

Glutathione peroxidase, an antioxidant selenoenzyme, can catalyze the reduction of hydroperoxides with the assistance of glutathione. It plays a crucial role in maintaining intracellular redox balance and protecting cell components against oxidative stress. However, similar to most natural enzymes, glutathione peroxidase has intrinsic disadvantages such as poor stability and sensitivity. To overcome these problems, numerous efforts have been devoted to the exploration of novel selenoenzyme mimics. Among others, GO nanocomposites were fabricated with excellent glutathione peroxidase‐like property to protect cells against oxidative stress. It was a highly efficient GO–Se nanozyme with excellent antioxidative property to protect against oxidative damage in intracellular microenvironment. These hybrid nanomaterials could catalyze the decomposition of H_2_O_2_ to harmless products effectively, and cell experiments confirmed their extraordinary ROS scavenging capacity [42].

To overcome the cell barrier problem, a designed mesoporous silica nanoparticle system with an attached denatured enzyme for enhancing cell membrane penetration was developed by Lin et al. Simultaneous delivery of two antioxidant enzymes, superoxide dismutase and glutathione peroxidase into HeLa cells shows synergistic efficiency of ROS scavenging. Enzyme–peptide conjugation provided endocytosis-independent cell uptake and escape from endosome while moving and aggregating along the cytoskeleton that would allow them to be close to each other at the same time, resulting in cellular oxidation protection. The two-enzyme delivery system shows a significant synergistic effect for protecting cells against ROS-induced cell damage and cell cycle arrest [43].

Enzyme-conjugated nanosystems are being used not only for cell/tissue protection but also to generate harmful species especially in targeted tumor therapies. Imaging-guided noninvasive tumor ablation [e.g., radiofrequency ablation, microwave ablation, laser ablation, high intensity-focused ultrasound ablation (HIFU)] has become a newly preferred treatment for certain cancers because of its high accuracy, cost-effective, and faster recovery advantages. However, HIFU surgery generally suffers from poor precision and low efficiency in clinical application, especially for cancer therapy. Contrast and synergistic agents for high intensity-focused ultrasound-guided cancer surgery have been developed by Liu et al. A hybrid catalytic nanoreactor that integrates dendritic-structured mesoporous organosilica nanoparticles and catalase was constructed for this purpose. This hybrid nanoreactor induces hydrogen peroxide (H_2_O_2_) decomposition continuously by releasing O_2_ bubbles as the echo amplifier for ultrasound imaging, which allows to enlarge ablated volume under HIFU exposure in the tissue-mimicking phantom due to the enhanced cavitation effect. This nanosystem further enables sustained contrast enhancement of ultrasound imaging within the H_2_O_2_-enriched tumor region, and high coagulative necrosis under HIFU. More importantly, this perfluorocarbon-free hybrid nanoreactor performs the tumor-sensitive theranostic function without HIFU pre-stimulation, which makes HIFU surgery much more precise and efficient for future clinical application [44].

Selenium acts as the active center of selenoproteins and induces changes in the activities of antioxidant enzymes, including glutathione peroxidase, superoxide dismutase, and catalase. Thus, catalase was immobilized on selenium nanoparticles by the formation of disulfide bonds. Intracellular reduction of disulfide bonds allows to induce the subsequent release of catalase, which catalyzes the decomposition of H_2_O_2_. The H_2_O_2_-depleting and O_2_-generating selenium NPs efficiently killed activated macrophages and quenched intracellular H_2_O_2_ and NO that were associated with inflammation. Macrophages have a pivotal role in chronic inflammatory diseases; therefore, imaging and controlling activated macrophage is critical for detecting and reducing chronic inflammation [45].

Apart from HIFU and potentially photodynamic selenium nanoparticles conjugated with a photosensitizer (rose bengal) and a thiolated chitosan (chitosan-glutathione), a new nanosystem with catalase loaded inside tantalum oxide nanoshells was fabricated [46]. The obtained catalase-carrying nanoparticles were then functionalized with polyethylene glycol. This bio-nanoreactor could efficiently improve tumor oxygenation by supplying oxygen through the decomposition of endogenic H_2_O_2_ in a tumor microenvironment, and thus synergistically enhance the efficacy of cancer radiotherapy by both depositing radiation energy within the tumor and overcoming hypoxia-induced radiotherapy resistance.

Immobilized catalase is intensively applied in various domains such as food industries, treatment of wastewaters, cosmetics, and pharmaceutical formulations to determine the hydrogen peroxide quantity or to remove excess H_2_O_2_. A review by Grigoras covers the updated information about the natural and synthetic materials based on polymers or low-molecular-weight compounds, organic or inorganic ones, used by researchers to immobilize catalase enzyme [47].

### Glucose oxidase

As glucose is a key nutrient and plays an essential role in supplying energy for tumor growth, glucose oxidase (GOX) was used to convert glucose into gluconic acid and hydrogen peroxide. Depletion of cell glucose reserves potentially provides an alternative strategy for cancer cell starving therapy. Hydrogen peroxide at high concentration can induce cancer cell death through hydrogen peroxide-dependent activation of apoptosis in tumor cells [48]. Therefore, the delivery of GOX to tumor is expected to consume intratumoral glucose for blocking the energy supply and simultaneously increasing the level of H_2_O_2_ for killing cancer cells [49, 50]. Native GOX is not stable due to the decomposition of flavin adenine dinucleotide, the catalytic center of GOX. Exogenous GOX also has drawbacks related to poor stability, short in vivo half-life, immunogenicity, and systemic toxicity. This is because GOX’s substrates, glucose and oxygen, are ubiquitous in the body, which results in the systemic generation of H_2_O_2_ and thus induces severe systemic side effects. To minimize the systemic toxicity of H_2_O_2_, aldehyde-functionalized GOX-loaded polymer nanogels were used for intratumoral administration in mice bearing C8161 melanoma tumors. GOX-polymer nanogels not only significantly enhanced the antitumor activity of GOX but also effectively reduced the side effects of GOX through the mechanism of limiting GOX presence to the tumor region [51]. Additionally, Fan et al. noted that the generated H_2_O_2_ can oxidize l-arginine (l-Arg) into NO for enhanced gas therapy. They constructed for the first time hollow mesoporous organosilica nanoparticles as a biocompatible/biodegradable nanocarrier for the co-delivery of GOX and l-Arg. The used of this nanosystem avoids the need for external excitation to obtain a remarkable H_2_O_2_–NO synergistic anticancer effect with minimal adverse effect [52].

### Horseradish peroxidase

On the basis of the observation that the production of ROS can initiate cellular damage and apoptosis, iron oxide nanoparticles with encapsulated horseradish peroxidase have been used to convert a benign prodrug, indole-3-acetic acid, to a toxic oxidized product. Indole-3-acetic acid reacts with horseradish peroxidase to form free radicals such as indolyl, skatole, and peroxyl. Simultaneously delivered horseradish peroxidase and indole-3-acetic acid exert toxicity due to high oxidative stress production abilities. Because tumor cells usually possess higher levels of scavengers for ROS, they may be more vulnerable to the combination of iron oxide NPs-encapsulated horseradish peroxidase and indole-3-acetic acid. Cancer cells have higher levels of ROS than normal cells, and if one further increases the ROS level, it leads to killing of cancer cell [53].

### l-Asparaginase

l-Asparaginase (ASNase II) is an effective antineoplastic agent used in the chemotherapy of acute lymphoblastic leukemia as a drug of choice. The enzyme can destroy asparagine-dependent tumors by degrading circulating l-asparagine and destroying malignant cells; however, in the native form, it is susceptible to proteolytic degradation by the proteases of the host organism. Production of its bioconjugates can increase its half-life and stability and decrease its immunogenicity. Ulu et al. [54] prepared biodegradable poly(methacrylic acid-co-methyl methacrylate) copolymer composite as a carrier matrix for the immobilization of l-ASNase. Bahreini et al. [55] reported the production, purification, and immobilization of the enzyme in chitosan cross-linked with tripolyphosphate nanoparticles. Thus, on the basis of findings of the presented works, both poly(methacrylic acid-co-methyl methacrylate) copolymer composite and chitosan nanoparticles can be used as the biocompatible matrix for l-ASNase immobilization to obtain a nanosystem characterized by increased in vivo half-life and prolonged release profile. Golestaneh and Varshosaz developed silica nanoparticles with 1-ethyl-3-(3-dimethylaminopropyl) carbodiimide hydrochloride as a crosslinking agent to l-asparaginase immobilization. A significant increase in the pH range of stability of NPs and a decrease in the Km value of the enzyme were obtained due to immobilization of this enzymatic drug on silica nanoparticles by using a cross-linker [56].

### Lysozyme

Recently, lysozyme extracted from a marine bacterium was demonstrated to inhibit angiogenesis and tumor growth in mice [57]. Lysozyme has also been shown to exhibit antitumorigenic activity in several other studies [58, 59]. Lysozyme adsorbed on biomimetic nanoporous silica nanoparticles was taken up into cells and even in the nucleus [60]. Self-assembled nanostructured lysozyme exhibited anticancer activity. These nanoparticles demonstrated excellent structural and functional stability of lysozyme in a wide range of pH and temperature with an acceptable level of protection against proteinase K digestion. The application of these NPs to MCF-7 breast cancer cells led to exhibited approximately 95% cell death within 24 h, involving some reactive oxygen species-based mechanism, with excellent hemocompatibility [61]. Self-assembled lysozyme-pectin nanogels were used as a carrier for the antitumor agent methotrexate by Lin et al. MTX-loaded nanogels had pH-dependent drug release profile. These nanogels could be effectively endocytosed by HepG2 cells, resulting in enhanced cancer cell apoptosis [62].

### Granzyme

Protein drugs with intracellular targets, such as granzyme B (GrB), represent an interesting class of enzymes that have demonstrated high antiproliferative activity in various cancer cells. Nanogels with a watery environment, high protein loading capacity, and excellent protein compatibility are particularly appealing for protein delivery. Chen et al. fabricated epidermal growth factor receptor and CD44 dual-targeted multifunctional hyaluronic acid nanogel for protein delivery to ovarian and breast cancers in vitro and in vivo. The modified nanogel was obtained by nanoprecipitation and photoclick chemistry from hyaluronic acid derivatives with tetrazole, GE11 peptide/tetrazole, and cystamine methacrylate moieties [63]. This constructed nanosystem was then loaded with granzyme B, which has high antiproliferative activity in cancer cells. The obtained nanogel showed fast protein release under a reductive condition. It exhibited excellent uptake in CD44- and EGFR-positive SKOV-3 ovarian cancer cells. Further, a GrB-loaded nanogel displayed enhanced caspase activity and growth inhibition in SKOV-3 cells. Interestingly, the studies of SKOV-3 human ovarian carcinoma and MDA-MB-231 human breast tumor xenografted in nude mice revealed that GrB-carrying nanogel at a low dose induced nearly complete growth suppression of both tumors without causing any adverse effects. The apoptotic mediators such as granzyme remained active following delivery and induced apoptosis in NIH-3T3 cells [63]. Granzyme A conjugated with arginine-functionalized gold nanoparticles provides a direct therapeutic application of intracellular protein delivery. GrA-mediated HeLa cell death was observed 24 h after the delivery of the nanosystem. Moreover, the delivered GrA-carrying NPs killed the cells through a caspase 3/7-independent pathway [64]. Nanoassembly-mediated Gr delivery may provide an efficient approach for intracellular protein therapy for cancer treatment.

Efficient and targeted cancer protein therapy in vivo by using bioresponsive fluorescent photoclick hyaluronic acid nanogels was described by Chen and colleagues. Two intracellular protein drugs, cytochrome c and granzyme B, were loaded into the nanogels with preserved bioactivity. Cytochrome c- and GrB-loaded hyaluronic acid nanogel could effectively target and release proteins to CD44-positive MCF-7 and A549 cancer cells, thereby yielding impactful antitumor effects. Remarkably, GrB-loaded hyaluronic acid nanogels exhibited complete suppression of tumor growth and minimal adverse effects in nude mice bearing subcutaneous MCF-7 human breast tumor and orthotopic A549 human lung tumor xenografts [65].

Granzyme B was delivered to CD44-overexpressing cancer cells on the self-assembled hyaluronic acid-epigallocatechin gallate conjugates, functionalized with linear polyethyleneimine (PEI). Hyaluronic acid was used for its ability to target CD44, which is overexpressed in many types of cancer cells, while epigallocatechin gallate, the main component of green tea catechins, was chosen for its ability to bind to proteins. Epigallocatechin gallate facilitated protein complexation through physical interactions, which led to the formation of stable nanogels. The obtained nanogel could kill CD44-targeted HCT-116 cancer cells by delivering GrB into the cytosol of these cells [66].

Enhanced drug and protein release can be achieved by designing a stimuli-sensitive polymer (polymer vesicles) with a diameter in the range of virus size and a natural watery core to internalize. To improve drug release features, many efforts have been directed to the development of smart polymers that release payloads in response to an internal signal such as cytoplasmic glutathione, enzyme, and endo/lysosomal pH or an external stimulus such as photo- and magnetic-based effects. GrB-loaded 2-[3-[5-amino-1-carboxypentyl]-ureido]-pentanedioic acid (Acupa)-decorated pH-responsive chimeric polymer induced effective apoptosis of prostate cancer cells (LNCaP) and had a long circulation time with an elimination phase half-life of 3.3 h in nude mice [23]. Furthermore, anisamide-decorated pH-sensitive degradable chimeric polymer was used by Lu et al. for the efficient delivery of granzyme to lung cancer cells [67].

### Caspase-3

Caspase-3 is a protein that plays an integral role in apoptosis. As most tumor cells circumvent this natural process, there is a significant interest in delivering functional caspase-3 as a therapeutic agent to target cancer cells. De Avila et al. showed that by using an ultrasound-propelled nanomotor approach and a high pH-responsive delivery strategy, caspase-3 could be delivered to gastric cancer cells. The authors first encapsulated the proapoptotic protein in the polymer Eudragit L30 D-55, which showed rapid dissolution above pH 5.5, thus ensuring safe arrival of the active enzyme to the intracellular environment while preventing caspase-3 release in the extracellular space. The polymer-encapsulated particles were subsequently coated on ultrasound-propelled gold nanowire motors. Uptake of the caspase-3-loaded nanosystem induced high levels of apoptosis, showing that large, charged enzymes could readily be administered to target tumors [68]. Caspase-3 was also delivered to osteosarcoma MG-63 cells by attaching it to poly(lactic-co-glycolic acid)-functionalized carbon nanotubes. Caspase 3-conjugated nanotubes could efficiently transduce and suppress cell proliferation [69].

### RNase A or protein kinase A

RNase A or protein kinase A, an endoribonuclease that specifically degrades single-stranded RNA at C and U residues, was attached to PEGylated GO through physical adsorption to induce apoptosis or cell proliferation. These composites were studied for protein delivery for the first time by Shen et al. GO-PEG was found to be biocompatible and efficient nanovector for protein delivery to cells. Moreover, GO-PEG could protect the adsorbed proteins from enzymolysis, which ensures that the biological functions of the delivered proteins are preserved. Furthermore, protein adsorbed onto GO was found to be stable for 6 h after exposure to trypsin [70]. To deliver ribonuclease A intracellularly using dextran nanogels for cancer treatment, Kordalivand et al. linked the protein covalently to the nanogel network through disulfide bonds, which are cleavable in the reductive cytosolic environment. Positively charged RNase A was electrostatically loaded in anionic dextran nanogels. The nanogels showed a fast and triggered release of RNase in the presence of glutathione [71].

### α-Galactosidase

Fabry disease is a genetic lysosomal storage disease caused by deficiency of α-galactosidase, the enzyme that degrades neutral glycosphingolipid transported to lysosome. Glycosphingolipid accumulation causes multi-organ dysfunction and premature death of the patient. Enzyme replacement therapy using recombinant α-galactosidase is the only treatment available for Fabry disease. To maximize the efficacy of treatment and to enhance cellular delivery and enzyme stability, a nanomaterial carrier was developed. Coupling of α-galactosidase to nanocarriers targeted to ICAM-1 allows to enhance its accumulation in the vasculature. The nanosystem constructed by Hsu et al. showed preferable circulation and biodistribution profile to the heart and kidneys. Therefore, enzyme load and carrier bulk-concentration of ICAM-1-targeted nanocarriers can be adjusted without negatively impacting targeting efficiency and yet providing enhanced enzyme delivery to Fabry target organs [72]. They used polystyrene particles coated with antibody [73]. Protein nanoparticles using human serum albumin and 30Kc19 protein, originating from silkworm, were also used to enhance the delivery and intracellular α-galactosidase stability. The nanoparticles exhibited enhanced cellular uptake and intracellular stability of delivered α-galactosidase in human foreskin fibroblasts. Additionally, they showed enhanced globotriaosylceramide degradation in Fabry patients’ fibroblasts [74].

## Targeting moieties

**Fig. 3:**
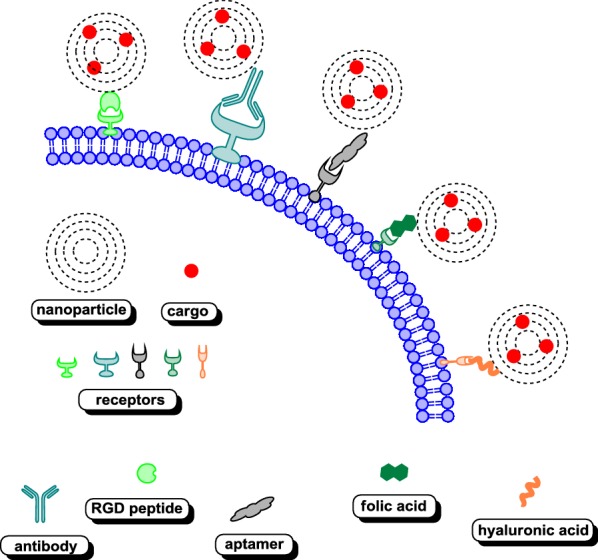
Potential receptor targeting strategies

The use of receptor targeting strategy for selective drug binding and internalization has been an attractive approach since early advances in cell biology, and it began to reveal built-in internalization pathways that enabled selective uptake of a particular component from the external environment. The currently used potential receptor targeting strategies are depicted in Fig. 3.

The use of these endogenous uptake pathways for drug delivery was tested, particularly in the context of findings related to the properties of tumor cells, which allowed identification of receptors that were overexpressed in rapidly proliferating cells. Interest in receptor targeting for selective uptake and internalization of drugs has expanded even further with the availability of sophisticated nanotechnology approaches to encapsulate drugs, thereby providing controlled release capacity and protection of macromolecules from degradation prior to reaching the site of action. Targeting moieties strategy is utilized as an anticancer drug as well as photosensitizers’ carrier.

### Folate receptors

**Fig. 4:**
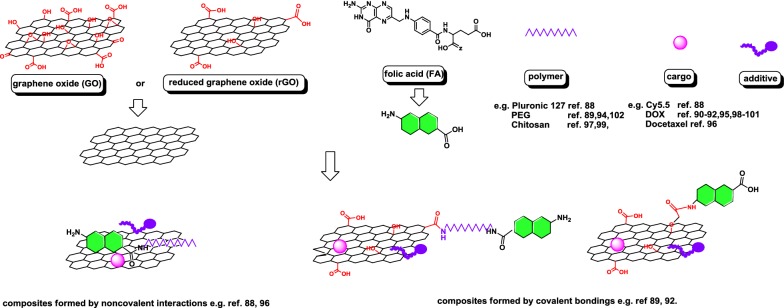
Schematic visualization of GO- or reduced graphene oxide-based folic acid receptor-targeted nanosystems

It has been reported that folate receptors (FR) are overexpressed on the surface of numerous cancer cells, including breast, ovary, lung, kidney, head and neck, brain, and myeloid cancers [75]. The overexpression of these receptors in tumor cells and their low and restricted distribution in normal tissues, along with the ability to observe tumor-promoting functions, together render FR an attractive therapeutic target. Considering this benefit, it is not surprising that folic acid has been used as a targeting ligand in various nanocarriers. There are numerous FA-targeted nanosystems constructed using GO, rGO, β-CD, magnetic NPs, and DNA and functionalized with different agents for use as drug nanocarriers and chemo-photothermal therapy facilitators and in radiotherapy and gene delivery [6, 10, 12, 14, 75–80]. Owing to the synergistic contribution of co-functionalized units, the constructed nanosystems can be used as a targeted delivery system to efficiently carry the drug molecules to specific sites with low toxicity to normal cells. An appropriate nanoparticle structure with the use of linkers susceptible to various factors, most important of which is tumor environment pH, improves drug release and allows to control their discharge [62, 67, 81, 82]. In vitro tests with folate receptor-targeted hybrid nanovehicles in a cancer cell line demonstrated exceptional cytotoxicity, high cellular uptake, and ability to induce apoptosis. A noteworthy finding is the increase in bioavailability and plasma circulation time of the carried drugs that was observed in an in vivo pharmacokinetic study. It was also demonstrated that cargo substances could be protected by the “shell” of functionalized nanomaterials during blood circulation [10, 83]. PEGylated folate was also used to deliver siRNA and miRNA, as described in the section “Gene delivery” [84–86]. Recent examples of FA-targeted nanomaterials are summarized in Table 1, and schematic visualization of GO- or reduced graphene oxide-based folic acid receptor-targeted nanosystems are presented in Fig. 4.

**Table 1:** Examples of folic acid receptor-targeted nanosystems

Type of nanomaterial	Functionalization	Cargo	Justification	Efficacy test	References
Abraxane^®^	FA	PTX, difluorinated curcumin	Targeted delivery system	SKOV-3, HeLa cells	[87]
Graphene oxide	Pluronic 127	Cy5.5	Fluorescence imaging	KB human epithelial mouth carcinoma cells KB tumor bearing mice	[88]
Graphene oxide	PEG	Fluorescein-labeled peptide with a recognizable sequence of DEVD, Camptothecin, curcumin, evodiamine, silybin	High-contrast caspase-3 activity imaging	HeLa cells HeLa tumor-bearing BALB/c nude mice	[89]
Graphene oxide	β-Cyclodextrin	DOX	Targeted delivery system	HeLa cells	[90]
Graphene oxide	PAMAM	DOX	Targeted delivery system	HeLa cells	[91]
Graphene oxide	Polyvinylpyrrolidone	DOX	Targeted chemo-photothermal therapy	HeLa cells	[92]
Graphene oxide		Chlorine e6	Targeted photodynamic therapy	MGC803	[93]
Graphene oxide	PEG, phospholipids	Resveratrol	Targeted chemo-photothermal therapy	MCF-7 cells Male athymic BALB/c mice	[94]
Reduced graphene oxide		DOX	Targeted delivery system	MDA-MB 231 cells	[95]
Reduced graphene oxide	Poloxamer 407	Docetaxel, irinotecan	Targeted chemo-photothermal therapy	MCF-7, HepG2 cells	[96]
Graphene oxide/Fe_3_O_4_	Thiolated chitosan	Coumarin 6	Targeted chemo-photothermal therapy	HeLa cells	[97]
Graphene oxide/Fe_3_O_4_		DOX	Targeted chemotherapy	SK3	[98]
Graphene oxide/Fe_3_O_4_	Chitosan	DOX	Targeted chemotherapy		[99]
Graphene oxide/gold		DOX	Targeted chemo-photothermal therapy	MCF-7 cells, HeLa cells Balb/c mice, New Zealand White rabbits	[100]
Lipid core nanocapsules		DOX, tanespimycin	Sequential delivery of DOX and tanespimycin, sensitize the cancer cells to the cytotoxic effects of DOX	MCF-7 cells	[101]
Poly(lactic-co-glycolic acid) (PLGA)	PEG, phospholipids	Boron-curcumin complex, gadolinium	Antiproliferative effect Neutron capture therapy, MRI imaging	IGROV-1 cells	[102]
PAMAM	PEG	Oxaliplatin	Targeted delivery system	SW 480, MSC	[103]
Polyphosphoester		DOX	Targeted delivery system	HeLa cells	[104]
Methoxy poly(ethylene glycol)-b-copolycarbonates		DOX	Targeted delivery system	HeLa cells, COS-7 cells	[105]
Chitosan		Hesperetin	Targeted delivery system	HCT15 cells	[106]
Chitosan	Oleic acid	Bromopyruvate	Targeted delivery system	HLF cells	[107]
Chitosan	Poly(2-dimethylaminoethyl methacrylate), casein coated iron oxide	5-Fluorouracil	Targeted delivery system	L929, MCF-7, MDA-MB-231 cells	[108]

Multidrug resistance (MDR) is an important issue that affects the successful chemotherapy using some anticancer drugs. MDR can exist intrinsically, but generally gradually develops during the chemotherapeutic treatment of initially sensitive cancer cells. Multiple mechanisms contribute to MDR, including inhibition of drug-induced apoptosis, activation of DNA damage repair mechanisms, and increased expression of the efflux pumps [109–111]. These efflux pumps recognize a broad range of drug molecules and rapidly and efficiently transport low molecular weight chemotherapeutic agents out of cancer cells. Currently, one of the strategies to overcome MDR is to use nanotechnology-based drug delivery systems. Because of their size, nanodrugs are internalized through endocytosis instead of passive diffusion across the cell membrane, thereby bypassing the drug efflux pumps responsible for MDR. The nanogels based on hydroxyethyl methacrylamide-oligoglycolates derivatives covalently loaded with doxorubicin and subsequently decorated with a folic acid-PEG conjugate constructed by Chen et al. could overcome multidrug resistance. Moreover, the acid-labile hydrazone bond between DOX and the methacrylamide polymeric network enabled pH-responsive drug release. The folate receptor-positive B16F10 melanoma cells showed specific uptake of the targeted nanogels. This demonstrated efficient internalization of nanogel, lysosomal trafficking, drug release, and nuclear localization of DOX. The folic acid-targeted nanogels allowed to avoid drug efflux pumps in DOX-resistant 4T1 breast cancer cells through upregulation of the folate receptor. This resulted in highly efficient killing of resistant cancer cells [112]. Ligustrazine also improves the sensitivity of multidrug-resistant cancer cells to chemotherapeutic agents. Cheng et al. synthetized folate-chitosan nanoparticles by combination of folate ester with the amine group on chitosan to use them as a delivery vehicle for ligustrazine. The obtained results showed adequate encapsulation efficiency, loading capacity, and release rate of ligustrazine. The cumulative release rate was approximately 95% at pH 5.0, which was higher than that at pH 7.4. Drug-carrying NPs were internalized into MCF-7 cells through FR [113]. Both biodegradable polymeric folic acid functionalized nanogels and ligustrazine chitosan folate receptor-positive tumor cell targeting drug delivery system have potential for treating multidrug resistant malignancies during cancer therapy.

### Antibodies and aptamers

Adjuvant targeting pattern recognition receptors have been intensively investigated for the induction of effective immune responses by therapeutic vaccines. In such strategies, functionalized nanoparticles are used that demonstrated targeted delivery features, when conjugated with a tumor-specific antibody or a molecule with strong affinity against a given receptor overexpressed in a tumor. Note that Toll-like receptor 4 ligand and lipopolysaccharides promote Th1 and CD8^+^ T cell responses through direct or indirect activation of dendritic cells [114]. Diverse NPs could be adopted as tumor-selective delivery vectors. The function of such vectors may be based on different mechanisms. Blocking interleukin 10 (IL10) production at the time of immunization to increase cytotoxic T-cell responses in antigen-experienced host is one of them [115, 116]. Ni et al. demonstrated that GO could absorb anti-IL10 receptor antibodies. Interleukin 10 is an anti-inflammatory cytokine that limits immune responses to both self and foreign antigens. The anti-IL10R antibodies adsorbed on GO were slowly released in a pH-dependent regime. GO-adsorbed anti-IL10R antibodies were active both in vitro and in vivo. GO decorated anti-IL10R antibodies were more efficient than free anti-IL10R antibodies in inducing lipopolysaccharide-stimulated CD8^+^ T-cell responses. GO-based antibody-modified NPs may be useful as an adjuvant for vaccination and ideal for delivering to tumor site and disrupting suppressive tumor environment [117].

Antibody-decorated nanoparticles are also used as suitable drug delivery systems targeted toward cancer cells. In Mekuria et al. study, PAMAM dendrimer (G4.5) physically loaded with DOX was conjugated with IL-6 antibody. Adequate cytotoxicity of the nanocarrier was obtained in in vitro study on HeLa cells. The cytotoxic effect was exerted due to delivery of nanovehicle through receptor-mediated endocytosis [118].

Tumor progression depends on angiogenesis. The formation of neovasculatures during cancer initiation, progression, and metastasis is a critical process [119, 120]. Targeting of angiogenic markers on the tumor vasculature can result in more efficient delivery of nanomaterials into tumor. Young et al. demonstrated efficient targeting of breast cancer metastasis in an experimental murine model with PEGylated GO, which was conjugated to a monoclonal antibody against follicle-stimulating hormone receptor. The follicle-stimulating hormone receptor has been confirmed to be a highly selective tumor vasculature marker, which is abundant in both primary and metastatic tumors [121]. It was observed that targeted GO NPs were more efficiently taken up by breast cancer lung metastasis nodules inside the lung than non-targeted GO conjugates. Furthermore, the vasculature accumulation of GO nanovehicle in the tumor was confirmed. In addition, these GO nanomaterials can be used as a drug carrier with satisfactory drug loading capacity (for doxorubicin, 756 mg g^−1^) [122].

Angiogenesis also affects integrins, particularly integrin αvβ3. The suppression of integrin αvβ3 with targeting antibodies, antagonists, or peptides has been shown to exert anticancer effects in various types of cancers [123]. A pH-responsive charge-reversal polyelectrolyte and integrin αvβ3 monoclonal antibody-functionalized GO complex was developed as a nanocarrier for targeted delivery and controlled release of DOX into cancer cells by Zhou et al. Integrin αvβ3 monoclonal antibodies and PEI were covalently bound to carboxylated GO and DOX was conjugated with anionic poly(allylamine hydrochloride). DOX-poly(allylamine hydrochloride) complex was loaded on the positively charged surface of GO/PEI by electrostatic forces. This constructed nanocarrier was characterized by highly efficient DOX load and its effective release under mild acidic pH environment. In vitro study on U87 MG cells, which overexpress integrin αvβ3, confirmed that using the constructed nanosystem, DOX was selectively transported into the targeted cancer cells. After being effectively released from the nanocarriers into the cytoplasm, DOX was transferred into the nucleus. Because of effective delivery and release of the anticancer drugs into the nucleus of the targeted cancer cells, high therapeutic efficiency was obtained [124].

Photoacoustic therapy for selectively killing tumor cells has shown promise for treating diverse tumors. Utilization of high optical absorption probes can help to effectively improve the efficiency of photoacoustic therapy. Yan and Qin reported a novel high absorption photoacoustic probe composed of indocyanine green attached to GO and conjugated with integrin αvβ3 monoclonal antibody. This kind of nanostructure was utilized to target the U87-MG human glioblastoma cells for selective tumor cell killing. Indocyanine green-attached GO particles exhibited a tenfold higher absorbance at 780 nm (its peak absorbance) than native GO. An indocyanine green modified GO could convert the absorbed light energy with high efficiency to acoustic wave under pulsed laser irradiation. The presented results confirm that the strong laser-induced wave from integrin-targeted indocyanine green-loaded GO could effectively induce U87-MG cell apoptosis thus showed high efficiency in killing tumor cells [125].

In the work of Wei et al., a photodynamic therapy together with drug delivery based on GO nanoparticles was developed to improve subcellular targeting and impact cancer cells. To achieve accepted assumptions, GO was modified with the integrin αvβ3 monoclonal antibody for tumor-targeting. To obtain phototoxicity feature, a pyropheophorbide-a conjugated with PEG was used to cover the surface of the GO nanomaterial. The nanosystem could efficiently target the αvβ3-positive tumor cells with surface ligand and receptor recognition. Integrin-targeted GO-based NPs were then endocytosed by the cells that escaped from lysosomes and transferred to the mitochondria. In the mitochondria, the nanosystem exerted phototoxicity by killing cells by mitochondria-mediated apoptosis [126].

Vascular endothelial growth factor (VEGF) receptors are the main signal transducers that stimulate endothelial cell migration and vessel formation. Orleth et al. described PEGylated liposomal doxorubicin targeted against VEGF2 receptor- and VEGF3 receptor-expressing cells by inserting anti-VEGF2 receptor and/or anti-VEGF3 receptor antibody fragments into the lipid bilayer membrane of DOX liposomal formulation. Selectively targeting vascular cells revealed a highly efficient reduction of tumor-associated vasculature. This leads to tumor starvation and pronounced reduction of tumor weight in vivo in the Rip1Tag2 mouse model of human cancer [127].

A novel antitumor drug delivery system, docetaxel-loaded oxidized single-wall carbon nanohorns with anti-VEGF monoclonal antibody as a target agent was constructed by Zhao et al. Docetaxel was absorbed onto the oxidized single-wall carbon nanohorns through physical adsorption or π–π interaction. PEG was non-covalently wrapped to the hydrophobic surface of the oxidized single-wall carbon nanohorns. The monoclonal antibody was then bonded to the PEG through amide bonds. The constructed nanohybrid exhibited enhanced permeability and retention effect and reduced the drug molecule uptake by the reticuloendothelial system. The nanovector augmented cytotoxicity in MCF-7 cell lines in vitro and provided higher antitumor efficacy in vivo in H22 xenograft tumor mouse model [128].

Non-Hodgkin lymphoma is one of the most common hematologic malignancies among adults, and the chimeric monoclonal anti-CD20 antibody rituximab is used as the first-line therapy for this cancer [129]. To improve rituximab cytotoxicity and increase therapeutic efficacy in lymphoma, Lou et al. conjugated GO with rituximab. The constructed nanocarrier demonstrated notably high affinity for CD20 moiety. Targeted binding of rituximab/GO conjugate to CD20-positive lymphoma cells induced cell death through an actin-dependent mechanism. In vivo, in a xenograft lymphoma mouse model, GO-based nanovector eliminates high-grade lymphomas. These findings represent the first demonstration of using GO-conjugated antibody as an effective cytotoxic therapy for human B-cell malignancies in the absence of chemotherapy [130]. In other studies, Jiang et al. utilized human anti-CD20 monoclonal antibody-targeted carbon nanoparticles, which simultaneously carried DOX as the chemotherapeutic agent. Results of an in vitro study demonstrated that the constructed polymeric liposome was an effective nanocarrier of both the monoclonal antibody and the chemotherapeutic agent and could be used to target chemotherapy to CD20-positive B-cells [131].

DNA aptamers are short single strands of DNA or RNA oligonucleotides that can readily self-hybridized with itself to show important tertiary structures. They can bind to cell surface receptors and enter cell targets with high specificity [78]. Aptamers, generated by the systematic evolution of ligands by exponential enrichment (SELEX) method, are short, artificial, single-stranded oligonucleotides that, similar to antibodies, interact at high affinity with their targets by recognizing a specific three-dimensional structure. Advantages of using aptamers over antibodies are that they have high specificity, low-molecular-weight, and easy surface immobilization through their functional groups. As they are easily reproducible, they are usually inexpensive and have a higher shelf life than antibodies [132, 133]. A number of aptamers capable of recognizing target receptors have been reported [78, 134–136]. The most commonly described aptamer MUC1 named S2.2 binds with high affinity to abnormally glycosylated mucin-1, a cell surface glycoprotein, which expresses in many cancers, such as breast, colon, lung, and ovarian cancers [137]. Increasingly more than one aptamer is used for targeting. Usually, the dual aptamer-functionalized nanomaterial contains ATP aptamer. Its presence could facilitate deconstruction of nanosystem in lysosome, which has high amounts of ATP. Because of these properties, one can achieve more effective and faster release of chemotherapeutics loaded on/into aptamer-decorated nanocarriers. It was proven that aptamer binds to its corresponding target with a higher binding constant relative to its complementary strand [138]. General principle of utilization of nanovector system usually consists of loaded anticancer drug-targeted delivery with ligands, which can specifically bind to tumor markers that are overexpressed on the surface of cancer cells but with relatively low expression or even no expression on normal cells and tissues. Moreover, few studies have attempted to load therapeutic aptamers on metallic NPs and demonstrate their functional activity in photodynamic therapy in vitro. As shown in Table 2, most studies to date have used carbon-based NPs for aptamer delivery, and only a few studies have used polymeric NPs.

**Table 2:** Examples of aptamers targeted nanosystems

Type of nanomaterial	Aptamer	Cargo	Justification	Efficacy test	References
Carboxyl-modified, streptavidin-coated magnetic beads	Extracellular peptide epitope of CD123 (ZW25)	DOX	Targeted chemotherapy	AML cells BALB/c mice	[139]
PEG	Integrin α6β4 (IDA), eIF4E (AS1411), MNK1 (apMNK2F)	Drug-free	Targeted chemotherapy	PC-3 cells, 4T1 cells BALB/c mice	[140]
Aptamer-based DNA diamond nanostructure	MUC1 ATP	Epirubicin	Targeted chemotherapy	C26 cells MCF-7 cells BALB/c mice	[141]
DNA nanoflowers	Sgc8 MUC1	Fluorescein, cyanine3 6-carboxy-X-rhodamine	Fluorescence imaging	MCF-7 cells	[142]
Gold nanorods	MUC1	DOX	Targeted chemo- and photothermal therapy	MCF-7 cells	[143]
PEG/gold nanorods	Ramos ATP	DOX	Targeted chemotherapy, fluorescence imaging	Ramos cells Ramos tumor-bearing nude mice	[144]
PEG-MnO	AS1411		MRI contrast agent	786–0 renal carcinoma, EA.hy926 cells BALB/c mice	[145]
GO	Sgc8c ATP	Drug-free	Targeted chemotherapy	Molt-4 cells	[146]
GO	MUC1 Vimentin (NAS-24)		Targeting apoptosis induction	MDA-MB-231 cells MCF-7 cells	[147]
GO	MUC1 Cytochrome c		Targeting apoptosis induction	MDA-MB-231 cells MCF-7 cells	[147]
GO/AuNP	MUC1	DOX	Targeted chemo- and photothermal therapy	MCF-7 cells, A549 cells	[148]
Mesoporous carbon NPs, polyacrylic acid, PEI	MUC1	DOX	Targeted chemotherapy	MCF-7 cells, A549 cells	[149]
MNPs	Sgc8c	DOX	Targeted chemotherapy	CEM, Ramos xenografted tumor mice	[150]
SPION (Au)	MUC1		Photothermal therapy MRI	HT-29 cells	[151]
Myristylated chitosan	Androgen-sensitive human prostate adenocarcinoma cells aptamer	DOX	Targeted chemotherapy	LNCaP cells	[152]
PLGA/chitosan	MUC1	Epirubicin	Targeted chemotherapy	MCF-7 cells BALB/c mice	[153]
PLGA/PEG	Platelatet-derived growth factor receptor β	PI3K-mTOR inhibitor	Deliver a multifunctional nanosystem to the brain	U87MG cells orthotopic cancer-bearing mice	[154]
PLGA/PEG	(HCC)-specific (TLS11a)	DOX	Targeted chemotherapy	HCC mouse liver cells	[155]
Liposomes/chitosan	Anti-EGFR	Erlotinib	Targeting apoptosis induction	H1975 cells PC-9 cells	[156]
QDs/Se	AS1411		Targeting apoptosis induction, fluorescence imaging	U87 cells U251 cells HeLa cells A375 cells Siha cells MCF-7 cells EJ cells	[157]
	ssDNA–GMT-3	DOX	Targeted chemotherapy	A-172 cells, MCF-7 cells	[158]

Aptamers are also considered as highly specific and efficient delivery agents for siRNA. A dual tumor-targeting siRNA delivery system combining pRNA dimers with chitosan nanoparticles was designed by Li et al. [159]. In this nanovehicle, folate-conjugated and PEGylated chitosan nanoparticles encapsulating pRNA dimers were used. In this system, tumor-targeting aptamer (FB4, an RNA aptamer specifically binding to the extracellular domain of a transferrin receptor expressed by MCF-7 cells) could deliver siRNA into the target cells through aptamer-mediated endocytosis in a specific and efficient manner. Both in vitro treatment of MCF-7 cells and in vivo treatment of MCF-7 tumor-bearing mice with aptamer-decorated chitosan NPs resulted in increased cellular uptake, stronger cell cytotoxicity, higher cell apoptosis, and more efficacious gene silencing. Higher accumulation of siRNA in the tumor site, stronger tumor inhibition, and longer circulating time were also observed [159] by using this nanocarrier. To deliver P-gp-targeted siRNA into breast cancer cells, Powell et al. constructed aptamer-functionalized nanoliposomes. Aptamer A6 was used that can bind to Her-2 receptors on breast cancer cells. In vitro tests indicated that because of enhancing the knockdown of the MDR gene, the aptamer-labeled P-gp siRNA-encapsulated NPs produced a greater cellular internalization and direct accumulation of drugs (doxorubicin) in the nuclear compartment of breast cancer cells, which allowed to overcome MDR phenomenon [160].

### Targeting proteins and peptides

#### Transferrin

Transferrin (Tf), a 78 kDa-monomeric serum glycoprotein, acts as an important transporter to deliver iron into cells by binding to the transferrin receptor and subsequently is internalized through receptor-mediated endocytosis. The transferrin receptor is overexpressed in tumor tissues. It has been shown that the cellular uptake of transferrin by tumors in rats is correlated with the proliferation activity of the tumor cells (i.e., the faster the tumor growth, the higher is the uptake of transferrin) [12]. Tf-conjugated NPs have thus been explored primarily for targeted chemotherapy as shown in Table 3.

**Table 3:** Examples of transferrin-targeted nanosystems

Type of nanomaterial	Cargo	Efficacy test	References
*O*-Carboxymethyl chitosan	DOX	HCT119 cells KB cells	[82]
PEG/chitosan	Paclitaxel	HOP-62 cells	[161]
Dextran-spermine NPs	Capecitabine	U87MG cells	[30]
Mesoporous silica	DOX	Huh7 cells	[162]
PLGA	DOX	PC3 cells	[163]
PLGA/SPION	Paclitaxel	MCF-7 cells U-87 cells	[164]
PEG	Curcumin, DOX	MCF-7 cells BALB/c nude mice	[165]
Pluronic 85/lipid	DOX	DOX resistance HL-60 cell line BALB/c mice	[166]
PEG/phosphatidyl micelles	Paclitaxel, tariguidar	SCOV-3TR cells A2780-Adr MDR cells	[167]
PEG-ylated vitamin-E/lipid core micelles	Curcumin	HeLa cells HepG2 cells	[168]
Tocopheryl-PEG succinate	Cisplatin	A549 cells A549 xenografts mice	[169]
PEG-hydrazone-glyceryl monostearate	Docetaxel and baicalein	A549 cells A549/DTX resistant cells	[170]
Polypyrrole	^131^I	U87MG cells	[171]
MoS_2_ nanoplates modified with block copolymer P(OEG-A)-b-P(VBA-co-KH570)	DOX	HepG2 cells	[172]
GO/PEI	Pt[10-methyldipyridophenazine] Cl_2_	MCF-7 cells	[173]
GO	Docetaxel	MCF-7 cells	[174]
PEG/GO	DOX	Murine C6 glioma cells BMVE cells Brain glioma-bearing rat model	[175]
Multi-walled carbon nanotubes	Docetaxel	A549 cells	[176]

Only few nanosystems were constructed to enhance photothermal therapy [164, 171, 172, 175], and one of the described nanovehicles can be used in radiotherapy [171]. An in vitro study on different types of cell lines indicated that Tf-targeted NPs were internalized by endocytosis and exhibited excellent cytotoxicity. NPs could be internalized through endocytosis by energy-dependent pathways. Overall, receptor-mediated endocytosis was the primary pathway [82, 161, 164, 165, 172, 176], but GO decorated with Pt, dextran-spermine biopolymer and micellar NPs cellular uptake involved clathrin-mediated endocytic and dynamin-dependent lipid raft-mediated pathways [30, 173]. Furthermore, micellar structure could be internalized via caveolin-dependent endocytosis and micropinocytosis. Specific uptake allowed lower concentration of loaded drugs to achieve the same therapeutic effect. Moreover, the drug cargo was usually released in a pH-dependent and sustained manner. Tf-decorated nanosystems also exhibited efficient tumor-targeted accumulation in a tumor-bearing nude mice model. They displayed strong antitumor effect.

Several micellar nanoformulations were evaluated on the basis of targeting efficiency, cellular association, cellular internalization pathway, and cytotoxicity for the reversal of chemotherapeutic agent resistance on miscellaneous multidrug-resistant cell lines [177–179].

Transferrin-targeting nanoparticles are also used to transfer genetic material. Xie et al. used the Tf-binding peptide conjugated to a cationic polymer branched PEI for selective delivery of siRNA into H1299cells. The Tf-bPEI conjugate exhibited siRNA condensation efficiency similar to unmodified bPEI. The obtained results revealed that Tf-bPEI selectively delivered siRNA to H1299 cells. Moreover, Tf-bPEI achieved efficient glyceraldehyde 3-phosphate dehydrogenase gene knockdown in H1299 cells [180]. Wei et al. also developed Tf-targeting effective siRNA delivery system by using layer-by-layer assembling of protamine/chondroitin sulfate/siRNA/cationic liposomes followed by T7 peptide modification. It was demonstrated that hybrid NPs could effectively deliver siEGFR into U87 glioma cells through TfR-mediated internalization and inhibit tumor proliferation by inducing EGFR downregulation. In vivo experiments showed that modified siEGFR-conjugated NPs could exhibit a higher accumulation in the brain tumor site and a higher therapeutic efficacy than non-targeted NPs [181].

#### VEGF

Angiogenesis plays a crucial role in facilitating malignant tumor growth. During angiogenesis, new blood vessels are formed, and this process involves endothelial cell migration, growth, and differentiation. Vascular endothelial growth factor is the crucial regulator of angiogenesis and acts through binding to the tyrosine kinase receptors VEGF receptor 1 (VEGF1 receptor, Flt-1) and VEGF2 receptor (KDR and Flk-1) [182, 183]. These receptors are overexpressed in tumor vasculatures. VEGF and its receptors, as major angiogenesis regulators, have recently been investigated as cancer therapeutic targets and tumor imaging agents [184, 185]. However, VEGF targeting moieties are being used more often by developing nanocarriers with siRNA or antibody than with VEGF itself.

A recent report described the conjugation of VEGF 121 to the PLGA nanoparticles. VEGF was conjugated on the surface of nanoparticles by the covalent coupling method. Synthetized VEGF–NPs were more associated to human umbilical vein endothelial cells (HUVEC) by binding to VEGF receptors. The in vitro cell proliferation test IC50 showed antiproliferative activity of paclitaxel-loaded VEGF–NPs due to their high cellular targeting of tumor cells [186]. PLGA nanoparticles have also used as the base to construct a VEGF-targeted nanosystem by Carenza et al. The PLGA nanocapsules contained bioactive VEGF165 in the inner core and superparamagnetic iron oxide nanoparticles embedded in the polymeric shell. Studies confirmed the augmentation of proliferation in human microvascular brain endothelial cell cultures. In addition, magnetic VEGF165-loaded PLGA nanocapsules could be moved and accumulated under an external magnetic field during targeting therapies and imaging [187].

Shi et al. developed surface engineering and in vivo tumor vasculature targeting of GO nanoconjugates in U87MG tumor-bearing mice, with VEGF121 as the targeting ligand. PEGylated GO was functionalized with 1,4,7-triazacyclononane-1,4,7-triacetic acid and reacted with VEGF 121-SH, resulting in VEGF 121-conjugated GO [188].

In the work of Goel, the use of uniform mesoporous silica nanoparticles for VEGF targeted positron emission tomography imaging and delivery of the anti-VEGF drug (sunitinib) in human glioblastoma murine models were proposed. Mesoporous silica nanoparticles were modified with polyethylene glycol and anti-VEGF ligand VEGF121 was covalently attached. Conducted studies confirmed the stability and VEGF-specific targeting ability of the constructed nanoconjugates. Results demonstrated that a significantly higher amount of sunitinib could be delivered to the U87MG tumor by targeting VEGF than that using non-targeted counterparts. In vivo PET imaging studies indicated almost threefold enhancement in the tumor accumulation of targeted mesoporous silica nanoparticles as compared to that using the non-targeted ones [189].

#### RGD peptide

Destroying the vasculature network of a tumor is an important new strategy for cancer therapy. Targeting of integrin enables to disrupt tumor neovasculature. Integrin αvβ3, a transmembrane receptor that binds to extracellular matrix proteins or other adhesion receptors on neighboring cells, is highly expressed on the surface of endothelial cells and facilitates the growth and survival of tumor neovasculature. RGD tripeptide (arginine–glycine–aspartic acid) is a structural recognition motif for cell surface integrins, including αvβ3 and α5β1. Nanoparticles decorated with RGD peptide were used as a targeted carrier to enhance the delivery of chemotherapeutics into tumor cells [190]. After internalization by receptor-mediated endocytosis this nanosystem disassembled under acidic condition in the presence of lysozymes and cell lysate, leading to sensitive controlled drug release. This type of targeting nanocarriers could also be utilized as a probe for optical imaging. Gao et al. fabricated a ‘nanobullet’ through the encapsulation of vinyl azide into αvβ3-targeting peptide-functionalized hollow copper sulfide nanoparticles. Hollow copper sulfide NPs exhibit a strong photoacoustic response and a high photothermal conversion efficiency. Owing to these moieties, the constructed nanohybrid was selectively internalized into integrin αvβ3-expressing tumor vasculature endothelial cells and dramatically increased the photoacoustic signals from the tumor neovasculature, thereby achieving a maximum signal-to-noise ratio at 4 h post-injection. Upon NIR irradiation, the local temperature increase triggered vinyl azide to release N_2_ bubbles rapidly. Subsequently, these N_2_ bubbles instantly explode to destroy the neovasculature and further induce necrosis of the surrounding tumor cells. A single-dose injection of azide-encapsulated, αvβ3-targeting peptide-functionalized hollow copper sulfide nanoparticles led to complete tumor regression after laser irradiation, with no tumor regrowth for 30 days. More importantly, high-resolution photoacoustic angiography, combined with excellent biodegradability, facilitated the precise destruction of tumor neovasculature by azide-encapsulated, αvβ3-targeting peptide-functionalized hollow copper sulfide nanoparticles without damaging normal tissues [191]. Examples of recently described RGD-targeted nanovehicle are summarized in Table 4.

**Table 4:** Examples of RGD-targeted nanovehicles

Type of nanomaterial	Targeting ligand	Justification	Efficacy test	References
GO/Pluronic F127	FA Cyclic RGD peptide	Photothermal therapy	KB cells	[192]
GO nanosheets/Poloxamer 188	Cyclic arginine–glycine–aspartic acid–tyrosine–lysine pentapeptides	Combretastain chemotherapy	HeLa cells	[193]
Graphene QDs	RGD peptide	DOX	U251 glioma cells	[194]
PEG/PLGA micelles	Stapled RGD	Paclitaxel chemotherapy BBB penetration	U87 cells HUVEC cells BALB/c nude mice	[195]
Lipid-coated PEG/PLGA	RDG peptide	Sorafenib and quercetin chemotherapy	HCC cells BALB/c nude mice	[196]
PAMAM	IL-6 antibody RGD peptide	DOX chemotherapy	HeLa cells	[118]
PEI-cyclodextrin-poly γ-glutamic acid	Gamma-glutamyl transpeptidase RDG peptide monensin	Targeting extrinsic apoptosis via TRAIL signalling	HCT8/ADR cells HCT8/ADR tumour-bearing BALB/c nude mice	[197]
PEG/poly(ε-caprolactone) micelles	Cyclic RGD peptide	DOX chemotherapy	U87MG cells Nude mice	[29]
Se/chitosan	RGD peptide	DOX chemotherapy	HUVEC cells C57BL/6 mice	[198]

RGD bearing nanoparticles could also be utilized as a nonviral tumor-targeting vector for siRNA transfer. Ren et al. prepared graphene oxide functionalized with RGD peptides and developed a GO-based VEGF-siRNA nanocarrier that can actively target tumors. The efficiency of gene silencing and tumor growth inhibitory activity of RDG-targeted siRNA-loaded NPs were investigated both in vitro and in vivo in an S180 tumor-bearing mice model. In vitro assays revealed that the expression levels of both VEGF mRNA and VEGF protein were downregulated. In vivo assays showed excellent accumulation of the NPs in tumor tissues [199]. Further, VEGF-siRNA/RGD nanodiamond was prepared by conjugating Arg-Gly-Asp-Ser peptide and VEGF-siRNA to nanodiamond delivery particles by Cui et al. VEGF-siRNA complexes were used in antiangiogenic gene therapy to inhibit tumor growth through the downregulation of the expression of vascular endothelial growth factor. The release assays indicated that the presence of RGDS peptide prolonged the release time of VEGF-siRNA complex and significantly decreased the expression of VEGF mRNA and protein. In HUVEC test, VEGF-siRNA/RGDS/nanodiamond decreased the formation of the tubes and exhibited no cytotoxicity [200]. For confirmation, a covalent conjugate of nanodots with RGDS and VEGF-siRNA was presented by the same group. In vitro, RGDS/VEGF-siRNA-decorated nanodots released and transferred VEGF-siRNA in a long-acting manner. Hybrid nanodots decreased the expression of VEGF mRNA and protein in HeLa cells. In vivo, RGD-targeted siRNA-loaded nanodots exerted gene silencing effect and inhibited tumor growth. FT-MS spectrum analysis revealed that the modified nanodots were mainly distributed in tumor tissue of the treated S180-xenografted ICR mice [201].

#### NGR peptide

In recent years, NGR peptide-based drug delivery systems containing the asparagine-glycine-arginine motif with potential imaging agents have attracted increasing attention. They are recognized by CD13/zinc-binding aminopeptidase N receptor isoforms. Decomposition of NGR peptides leads to the release of Asp. This motif is recognized by RGD-binding integrins that are characteristic for tumor metastasis. CD13 is a type II transmembrane glycoprotein that is present in many solid tumors; it promotes tumor adhesion, invasion, migration, chemoresistance, and angiogenesis [202]. A worthwhile finding is that CD13 is expressed entirely in tumor vasculatures, but not in normal vessels. High expression of CD13 has also been detected in glioma and tumor-associated neovascularization [203]. Peptides binding to CD13 and RGD-binding integrins provide tumor-targeting, which can be used for dual-targeted delivery of anticancer drugs.

Graziadio et al. studied structural requirements for effective CD13 targeting with carboxyl Arg-Cys-COOH terminus or amino H_2_N-Cys-Asn terminus of cyclic NGR peptides. Carboxyl-terminus functionalized cNGR peptides showed better affinity for porcine aminopeptidase N receptor and greater capacity to target and be internalized into CD13 receptor-expressing cells than amino H_2_N-Cys-Asn terminus ones [204].

Enyedi et al. synthesized small cyclic NGR peptide-daunorubicin conjugates by using NGR peptides of varying stability with or without thioether bond. The cytotoxic effect of the novel cyclic NGR peptide-daunorubicin conjugates were examined in vitro in the CD13-positive HT-1080 (human fibrosarcoma) cell line. Results confirmed the influence of the structure on the antitumor activity and dual-acting properties of the conjugates. Attachment of the drug through the enzyme-labile spacer to the C-terminus of cyclic NGR peptide resulted in higher antitumor activity on CD13-positive cells [205].

NGR-conjugated PEG-CdSe/ZnS quantum dots were synthesized and characterized by Huang et al. PEG-based long circulation, CdSe/ZnS quantum dots-based nanoscale and fluorescence, and NGR peptide-based specific CD13 recognition were achieved with this system. The conjugated NGR peptides could recognize CD13 epitopes expressed on tumor cells. The described nanomaterials with non-toxic concentrations could cross the BBB and target CD13-overexpressing glioma and tumor vasculature in vitro (on primary rat brain capillary endothelial cells) and in vivo (on SD rats bearing C6 brain tumor), thereby contributing to fluorescence imaging of this brain malignancy [206].

A new drug targeting system for CD13-positive tumors, based on ultrasound-sensitive phosphatidylcholine nanobubbles and cell-permeable peptides has been developed by Lin et al. The cell-permeable peptides–doxorubicin conjugate was captured in the asparagine–glycine–arginine peptide-modified nanobubbles. Over 85% of the encapsulated cell-permeable peptides-doxorubicin conjugates were released from the nanobubbles in the presence of ultrasound according to in vitro study results. Cell experiments also showed high uptake of the conjugate in human fibrosarcoma cells (CD13-positive HT-1080). The constructed nanobubbles with ultrasound treatment exhibited an increased cytotoxic activity than those without ultrasound. In nude mice xenografted with HT-1080 cells, NGR-targeted nanobubbles with ultrasound showed a high tumor inhibition effect and long median survival time [207].

Taking advantage of their tumor-targeting property, NGR motif-containing peptides are used as cell penetration peptides to target the delivery of siRNA to trigger RNAi. In the study of Huang, a bi-functional (cyclic NGR motif and its isomerization product) peptide was designed and tested for siRNA delivery in vitro and in vivo. Assays revealed that the peptide/siRNA complexes were effectively internalized by MDA-MB-231 cells (a breast carcinoma cell line). Gene silencing assays indicated that anti-Lamin A/C siRNA delivered by bi-functional peptide silenced gene expression in MDA-MB-231 and HUVEC cells. Moreover, the siRNAs were efficiently delivered into tumor tissues and localized around the nuclei, as revealed by in vivo imaging of C57BL/6 mice and cryosection examination [208].

### Polysaccharides

Hyaluronic acid (HA) has unique physicochemical properties such as biodegradability, biocompatibility, nonimmunogenicity, nontoxicity, and the presence of numerous functional groups (–COOH, –OH) for modification or functionalization, which make it an ideal biomaterial for various biomedical applications [209]. HA can bind specifically to CD44 motif overexpressed on the surface of tumor cells that makes it suitable as a target molecule. CD44 is a family of cell surface glycoproteins that are involved in cell–cell interactions, cell adhesion, and migration in many malignant tumors. Overexpression of CD44 on cancer cells augments tumor aggressiveness by increasing adhesion to its extracellular matrix ligand HA. The overexpression of CD44 on cancer cells redirects a number of oncogenic pathways including the central Pi3K/Akt/NF-kB pathway, leading to cancer progression and malignancy [210]. Moreover, many studies showed that tumor cells produce a large quantity of hyaluronidase (HAase). HA-based nanoparticles can be selectively disintegrated by HAase [211, 212]. HA has become an attractive ligand for intracellular delivery to CD44-overexpressing tumors. Owing to this, HA has been applied to functionalize nanomaterials for both enhancing stability and drug targeting property and reducing undesired toxicity in vitro and in vivo. A noteworthy observation is that the additional targeting moiety (methotrexate, FA, and RGD peptide) utilized promoted internalization not only by tumor cells with CD44 receptor overexpression. This approach allows to take advantage of synergistic growth inhibition of tumor cells. HA-targeted nanovectors constructed in recent years are described in Table 5.

**Table 5:** Examples of hyaluronic acid-containing nanovectors

Type of nanomaterial	Cargo	Justification	Efficacy test	References
GO/RGD	DOX	Chemotherapy	SCOV-3 cells	[213]
Carbon dots/PEI	Plasmid pGL-3 and pEGFP-N1	Fluorescent imaging	HeLa cells	[214]
Carbon dots/PEI/mesoporous silica	GOX HAase DOX	Chemotherapy Fluorescent imaging	A549 cells	[51]
Octadecylamine conjugate	Methotrexate Curcumin	Chemotherapy Fluorescence imaging	HeLa cells	[215]
PLGA	Docetaxel Sulforaphan	Chemotherapy	MCF-7 cell BALB/c nude mice	[216]
PLGA	Salinomycin Paclitaxel	Chemotherapy	MCF-7 cell MDA-MB-231 cells	[217]
PLGA	Paclitaxel Curcumin	Chemotherapy	MCF-7 cell BALB/c nude mice	[218]
PLGA	Paclitaxel	Chemotherapy	MDA-MB-231 cells	[219]
PLGA/PEG	IC87114-quinazolinone purin	Chemotherapy	MiaPaca-2 cells	[220]
PLGA	Chlorine e6, Gd^3+^	Photodynamic therapy, MRI	A549 cells	[221]
Arginine based poly(ester amide)s	Chlorine e6	Photodynamic therapy	MDA-MB-231	[222]
PLGA/tocopherol	Docetaxel	Chemotherapy	A549 cells BALB/c nude mice	[223]
Magnetic Prussian blue/CuInS2/ZnS quantum dots/BSA		Fluorescence imaging MRI	HeLa cells BALB/c nude mice	[224]
HA	IR-780-NH_2_ dye	Fluorescence imaging	MB-49 cells C57BL/6 mice	[212]
Heptamethine cyanine-conjugated HA micelles	IR-808 dye	Fluorescence and photoacoustic imaging	A549 cells BALB/c nude mice	[170]
Diketopyrrolopyrrole derivative		Photodynamic therapy	HCT-116 cells HCT-116 tumor-bearing mice	[225]
Maillard reaction-based conjugates of HA and BSA micelles	Paclitaxel Imidazoacridinones	Chemotherapy	SKOV-3 cells	[226]
HA-ceramide/Soluplus	Resveratrol	Chemotherapy Fluorescence imaging	MDA-MB-231 BALB/c nude mice	[227]
HA	10-Hydroxycamtothecin	Chemotherapy	MDA-MB-231cells HT29 cells A549 cells HepG2 cells MDA-MB-435 cells Athymic nude mice	[228]
HA nanogel	Cytochrome c	Chemotherapy	MCF-7 cells Nude mice	[229]
HA nanoemulsion	Paclitaxel	Chemotherapy	Nude mice	[230]
HA-tocopherol succinate micelles	Paclitaxel	Chemotherapy	B16F10 cells and L-02 cells B16F10 tumor-bearing C57BL/6 mice	[231]
HA functionalized liposomes	DOX	Chemotherapy	MG63 cells Balb/c nude mice	[232]
HA modified liposomes/RGD	DOX	Chemotherapy	B16F10 cells MCF-7 cells C57BL/6 mice	[233]
HA functionalized liposomes/magnetic NPs	Docetaxel	Chemo-photodynamic therapy	MCF-7 cells	[234]
SPIONs			A459 cells	[235]
HA/deoxycholic acid/histidine micelles	Paclitaxel	Chemotherapy	MCF-7 cells Nude mice	[236]
HA/tocopherol micelles	Paclitaxel	Chemotherapy	SCOV-3 cells	[237]
Chitosan	5-FU	Chemotherapy	A549 cells	[238]
Chitosan	Docetaxel	Chemotherapy	MCF-7 cell	[239]
Mesoporous silica	Paclitaxel	Chemotherapy	MCF-7 cell Balb/c nude mice	[240]
Mesoporous silica/RGD	Chlorambucil	Chemotherapy	SCOV-3 cells	[241]
Mesoporous silica	DOX	Chemotherapy	HeLa cells	[242]

Hyaluronic acid nanoparticles can also be used to deliver an RNA/DNA cargo to cells overexpressing HA receptors such as CD44. Hwang et al. developed a fluorescence-sensitive theranostic nanoplatform by using hyaluronic acid-conjugated GO, which is capable of simultaneously sensing oncogenic miR-21 and inhibiting its tumorigenicity. Cy3-labeled anti sense miR-21 peptide nucleic acid probes loaded onto hyaluronic acid-conjugated GO specifically targeted CD44-positive MBA-MB231 cells and showed fluorescence recovery by interacting with endogenous miR-21 in the cytoplasm of the abovementioned cells. Decreased proliferation and reduced migration of cancer cells and the induction of apoptosis were achieved by knockdown of endogenous miR-21 by hyaluronic acid-conjugated GO. Fluorescence signals observed in vivo in tumor-bearing mice were high and long lasting [243].

Encapsulation of pDNA lipoplex into copolymers consisted of high-molecular-weight hyaluronic acid-conjugated with PLGA-PEG allowed to construct a micelle plasmid DNA delivery system. The specific and strong binding of HA to CD44 receptors followed by endocytosis resulted in high transfection of the constructed micelles in CD44-positive MDA-MB-231 and MCF-7 breast cancer cells [244].

Gu et al. developed a ternary nanosystem based on HA-coating PAMAM for targeted gene delivery to CD44-positive tumors. A novel reducible hyper branched poly(amido amine) was attached to plasmid DNA. HA nanovectors were fabricated by coating HA on the surface of the PAMAM/pDNA complexes core through electrostatic interaction. The ternary nanovehicle could efficiently protect the condensed pDNA from enzymatic degradation by DNase I, and HA could significantly improve the stability of nanomaterial in the sodium heparin solution or serum in vitro. HA also significantly decreased the cytotoxicity of the nanosystem due to the negative surface charge. Moreover, HA-modified nanovehicle showed higher transfection activity than the non-targeted one in B16F10 melanoma cells because of the active recognition between HA and CD44 receptor. In vivo results indicated that the interaction between HA and CD44 receptor dramatically improved the accumulation of the nanovector in CD44-positive tumor, leading to high gene expression [245].

## Gene delivery

**Fig. 5:**
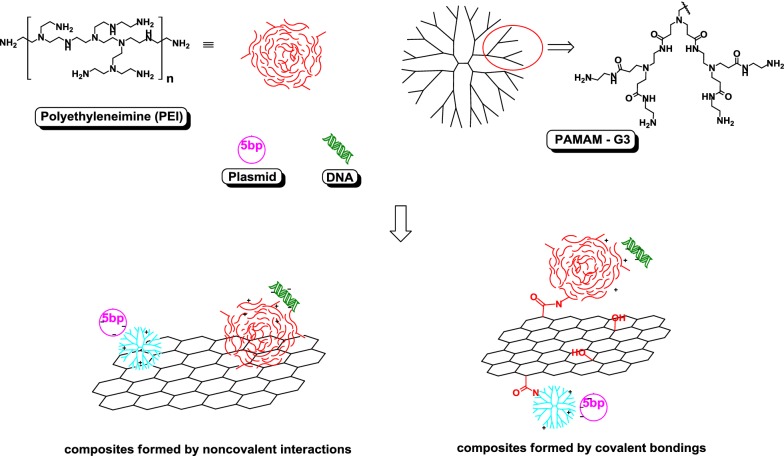
Schematic visualization of graphene oxide/cationic polymers gene delivery vectors [250, 251]

The safe and effective delivery of target genes to a specific site is substantially important for successful gene therapy. In general, the gene therapy consists of curing cancer by re-establishing a mutated gene or replacing a diseased one. Because of the risks associated with the usage of viral vectors, researchers are now exploring nonviral gene vectors. Among all kinds of gene carriers, cationic polymeric transporters (including polyethyleneimine, polyamidoamine dendrimers, polylysine, chitosan, and modified cationic polymers) for the delivery of therapeutic gene have received growing interests because of their improved high transfection efficiency and the relative safety. Schematic visualization of graphene oxide/cationic polymers gene delivery vectors is depicted in Fig. 5.

The presently used nonviral gene vectors are positively charged, linear or branched polymers that can form nanoscale complexes with nucleic acids, leading to their protection, cellular delivery, and intracellular release. The complexes (cationic polymers/nucleic acid) are based on electrostatic interactions, which are formed between the positively charged cationic polymers and negatively charged nucleic acid. PEI has been used as a gold standard in both in vitro and in vivo gene delivery agent, given its high buffering capacity for the endosomal escape of the delivered genes [246, 247]. For a summary of all polymer-based nucleic acid nonviral vectors characterized to date, the reader is referred to the following excellent reviews [81, 83, 248]. A few examples are cited below to enable understanding of potential nonviral therapeutic gene delivery agents.

### DNA

In the work of Feng et al., graphene was successfully used for the first time as a non-toxic nanovehicle for efficient gene transfection. GO was bounded with cationic polymers, PEI with two different molecular weights of 1.2 kDa and 10 kDa. Both the complexes were stable in physiological solutions. The GO–PEI-10 kDa complex exhibited significantly reduced toxicity to the treated cells compared to the native PEI-10 kDa polymer. Enhanced green fluorescence protein gene (EGFP) transfection with PEI-1.2 kDa appears to be ineffective; however, high EGFP expression was observed in HeLa cells using the corresponding complex consisting of GO and PEI 1.2 kDa as the transfection agent [249]. Apart from this, plasmid DNA encoding luciferase was successfully transfected by Chen et al. using the GO-PEI complex. Intracellular tracking of Cy3-labeled pGL-3 indicates that PEI-GO could effectively deliver plasmid DNA into cells and be localized in the nucleus [250]. An efficient gene delivery system based on GO functionalized with a non-toxic linear PEI was also reported by Tripathi et al. Linear PEI-grafted GO conjugates, prepared in the study with net positive charge density, efficiently condensed pDNA (pGFP) and delivered it to the internal compartment of the cells. Linear PEI–GO/pDNA formulation could exhibit transfection efficiency several folds higher than those of native linear PEI and branched PEI in HeLa and Hec293 cells [251].

Plasmid DNA encoding luciferase was also delivered using branched PEI-conjugated GO in a study conducted by Kim et al. The authors suggested that the amount of PEI conjugates on GO is a crucial factor to determine the capacity for plasmid DNA complexation. Branched PEI underwent complete complexation with pDNA at a low N/P ratio of 5. Plasmid DNA-complexed with branched PEI conjugated GO showed efficient transfection of HeLa and PC-3 cells [252]. Linear, 60-kDa PEI has also been used for the transfection of plasmid DNA encoding green fluorescent protein in cells and a zebrafish embryo model by Zhou et al. Plasmid DNA could be transfected into H293T cells and human osteosarcoma U2OS cell lines with up to 95% efficiency and 90% viability. This study also reported that the transfection efficiency of linear PEI alone was lower and cytotoxicity was higher than that of GO modified by the linear PEI nanosystem. The best transfection efficacy was achieved with polyethyleneimine-grafted ultra-small GO with the N/P ratio of 9.4. PEI complexed with ultra-small GO allowed gene transfection protocols to last for up to 72 h without increase in cell death. Moreover, microinjection of zebrafish embryos with plasmid DNA complexed to linear PEI-conjugated GO resulted in the expression of green fluorescent protein with low toxicity [246].

GO functionalized with covalently conjugated PEI through amide linkage with two polymers, i.e., GO–PEG–PEI and GO–PEI–PEG, were used as gene vectors by Feng and Kim [253, 254]. Reduced GO was decorated with branched PEI using hydrophilic PEG as a spacer. Low molecular-weight PEI linked with PEG-modified rGO could form complexes with plasmid DNA encoding the luciferase gene. The 1.8-kDa branched PEI and PEG-modified rGO supports expression of the encoded genes in PC-3 and NIH3T3 cells. This kind of nanostructure allowed to use photothermal effects to facilitate transfection, showing that irradiation of cells with NIR enhanced transfection efficiency [254].

Another platform that has been tested for the delivery of plasmid DNA is polyamidoamine dendrimers (PAMAM) and oleic acid-functionalized graphene. In this study non-viral nanovectors were synthesized by oleic acid adsorption on GO followed by covalent linkage of PAMAM dendrimers. The use of graphene-oleate-PAMAM carrier resulted in mammalian cell type- and dose-dependent cytotoxicity. The tested nanosystem was biocompatible to HeLa cells, and the cellular viability was maintained at about 80% when the hybrid concentration was up to 100 μg mL^−1^; however, it exhibited cytotoxicity to MG-63 cells at concentration higher than 20 μg mL^−1^. Moreover, the graphene-oleate-PAMAM possessed good biocompatibility and greatly improved the gene transfection efficiency of green fluorescent protein (18.3%) [255].

Plasmid DNA encoding luciferase has also been delivered using the hydroxylated PAMAM attached to *S*-methyl-l-cysteine via an acid-labile β-thiopropionate ester bond, followed by modification with folic acid through a polyethylene glycol linker. The nanovector could condense DNA (green fluorescent protein gene) to form spherical nanoparticles with particle size of ~ 200 nm. The DNA release rate was 95.8 ± 3.3%. Modified PAMAM/DNA complex was less cytotoxic to KB and HepG2 cells and exhibited higher gene transfection efficiency than native PAMAM/DNA. The uptake assays showed that polyplex entered KB cells within 0.5 h through folate receptor-mediated endocytosis and escaped from endosomes within 2 h. In addition, polyplexes displayed long circulation time along with excellent targeting of tumor sites in vivo in tumor-bearing nude mice [256].

Chitosan derivatives have been successfully introduced onto GO to load the plasmid DNA. Folate-conjugated trimethyl chitosan/GO nanocomplexes were prepared by Hu et al. This approach allowed co-delivery of genes and the chemotherapeutic drug DOX. The higher uptake level in HeLa cells with folate receptor than in A549 cells proved the targeting ability of the nanocarrier, suggesting that polyplexes might be internalized through FA receptor-mediated endocytosis. Both DOX and pDNA could be effectively loaded into the nanovector. The loading capacity of DOX reached 30.9% and migration of pDNA could be completely retarded. The accumulative release of pDNA was 31.1% at 72 h. In vitro folate/trimethyl chitosan-modified GO at concentrations up to 80 mg L^−1^ after incubation for 48 h did not reduce the viability of HeLa cells [257].

Chitosan has also been used as an anchor for surface modification of graphene nanotubes with other targeting ligands. Liu et al. utilized the multiwalled graphene nanotubes of different length and functionalized them with chitosan-folic acid nanoparticles. The constructed NPs could deliver the plasmid DNA with enhanced green fluorescent protein into HeLa and MCF-7 cells where the exogenous GFP gene was expressed. The shorter nanotubes exhibited higher cytotoxicity than the longer ones but also had greater gene transfection ability. Moreover, the surface functionalization of multiwalled nanotubes with chitosan and folic acid improved transfection efficiency and decreased cytotoxicity. The enhanced green fluorescent protein gene transfection efficiency of graphene nanotubes-based NPs was approximately 4.1%. The reported NPs show little effect on cellular viability when the concentration was up to 250 g mL^−1^ [258].

Graphene and carbon multi-walled nanotube nanocomposites were designed by Hollanda et al. for transfection of pIRES plasmid conjugated with green fluorescent protein. NIH-3T3 and NG97 cell lines could phagocytize graphene nanotube nanocomposites complexed with pIRES plasmid conjugated with the GFP gene. This nanocomposite was selectively cytotoxic. At high doses, it reduced the viability of NG97 cells, while it had no effect on NIH-3T3 cells [259]. In other recent studies, Nia et al. successfully used polyethyleneimine conjugates of single-walled carbon nanotube containing bioreducible disulfide bonds for the transfection of plasmid DNA with green fluorescent protein [260].

Several studies have investigated nongraphene-based nanomaterials as gene delivery agents. To improve gene delivery efficiency, Davoodi et al. incorporated pCMV-p53 plasmid DNA into core–shell microparticles, where the core and shell layers were loaded with nutlin-3a and β-cyclodextrin/chitosan/p53 nanoparticles, respectively. It was found that controlled and sustained release of both agents (nutlin-3 and p53) from the microparticles synergistically enhanced the antiproliferative efficacy through the continuous overexpression of p53 and caspase 3 proteins over 5 days. Additionally, nutlin-3a induced excessive oxidative stress in cancer cells. Moreover, a secondary apoptotic pathway by nutlin-3a was induced by the overproduction of ROS with irreversible destructive effects on subcellular organelles such as the nucleus and mitochondria [261]. Wang et al. also used core–shell-structured polyplex to transfer the fluorescent probe YOYO-1-labeled DNA with high transfection efficacy and low toxicity. The core–shell-structured polyplex was constructed by a core complex of G8 polyamidoamine dendrimer-condensed plasmid DNA further surrounded by a shell of low-molecular-weight PEI. Because of the lack of tertiary amine groups in their structures, PEI promoted DNA release from the polyplex. Furthermore, the hydrophobic nature of PEI might facilitate the escape of the polyplex from the endosome [262].

An injectable supramolecular hydrogel system based on the polypseudorotaxane formation between α-cyclodextrin and cationic methoxy-poly(ethylene glycol)b-poly(ε-caprolactone)-b-poly(ethylene imine) has been studied for the delivery of plasmid DNA. In this study, biodegradable and cationic triblock copolymer of methoxy-poly(ethylene glycol)-b-poly-(ε-b-caprolactone)-b-poly(ethylene imine) with PEI cationic segment was synthesized to condense Bcl-2 converting Nur77 gene for the formation of polyplex. This copolymer could in situ form solid state hydrogels upon the addition of cyclic α-cyclodextrins, because of the polypseudorotaxane formation between PEG segments and α-CDs. For the first time, a supramolecular hydrogel with the Nur77 gene polyplex with sustained release ability could effectively inhibit the growth of Bcl-2-overexpressing therapy-resistant tumor in vivo [263].

Zhao et al. designed comb-shaped polycations with neutral dextran as the main chain and folate-coupled bio reducible poly(urethane amine) as a non-viral vector for gene plasmids (pCMV-GFP and pCMV-Luc). The terminal groups of the poly(urethane amine) nanosystem were modified with dextran and folate. These comb-shaped polycations could attach genes to colloidal stable polyplexes and liberated them in response to a reductive intracellular environment. It was found that dextran-modified vector has excellent transfection efficiency against SKOV-3 ovarian cancer cells. Polyplexes were intravenously administered to nude mice with SKOV-3 tumors, affording a higher level of gene accumulation in the tumor than by polyplexes lacking folate. Moreover, in vivo gene therapy using a small hairpin RNA silencing VEGF and tested polyplexes led to the significant growth inhibition of SKOV-3 tumors with negligible systemic toxicity [264].

Another platform that has been tested for the delivery of plasmid DNA is succinoyl tetraethylene pentamine-histidine oligomer-based nonviral gene delivery system. The plasmid DNA encoding inhibitor of growth 4 (pING4) was transfected. Interleukin-6 receptor-binding I6P7 peptide was used at the same time for targeted drug delivery in glioma therapy. In vivo experiments indicate that the modification of NPs could enhance the BBB-crossing and glioma-targeting efficiency. Intravenous administration of I6P7 peptide NPs encoding ING4 reduced the growth of orthotopic glioma without any obvious sign of toxicity to normal tissues [28].

### RNA

In addition to pDNA, small interfering RNA (siRNA) with therapeutic functions may also be delivered by all types of graphene-nanopolymer complexes into cancer cells for potential gene therapy, which could be further combined with graphene-based chemotherapy and photothermal therapy [249–254]. RNA interference (RNAi) treatment, belonging to the post-transcriptional gene silencing process, is a promising and effective method for gene therapy in cancer treatments. Small interference RNA (siRNA) plays an indispensable role in gene silencing. However, naked siRNA has difficulty in crossing the cell membrane and can easily be deactivated by enzymolysis. Developing a new delivery system to circumvent complex extra- and intracellular barriers for successful translation is the gene-targeting technology that has great potential.

Yin et al. utilized multi-functionalized PEG, poly-allylamine hydrochloride, monolayer GO as a gene delivery system to efficiently co-deliver small interfering RNAs targeting the HDAC1 gene and the G12C mutant K-Ras gene to specifically target MIA PaCa-2 pancreatic cancer cells. Because of the high expression of folate receptor in MIA PaCa-2 cells, targeted delivery can be achieved by conjugation of FA with GO through receptor-mediated endocytosis. The clarification of the dual gene silencing effects indicated the inactivation of both the HDAC1 and K-Ras genes, hence causing apoptosis, cell proliferation inhibition, and cell cycle stop in treated MIA PaCa-2 cells. Transcriptome analysis further confirmed the inhibition mechanism of HDAC1 and K-Ras knockdown. The synergistic combination of gene silencing and NIR light thermotherapy showed significant anticancer efficacy, inhibiting in vivo tumor volume growth by > 80%. Furthermore, GO was used in the mouse model within a reasonable period of time without any obvious side effects [265]. Functionalized GO for the targeted intracellular delivery of hTERT siRNA was also prepared. This was possible due to conjugation of GO with PEG and folic acid, followed by the loading of siRNA. It was found that the conjugate could target HeLa cells in vitro and the transfected hTERT siRNA could efficiently inhibit the protein expression level and mRNA level [84].

A novel nanosystem of GO functionalized with polyethylene imine and polyethylene glycol was successfully synthetized and used to deliver plasmid-based Stat3 siRNA by Yin et al. The used plasmid was transfected into B16 cells with high efficiency and Stat3-related gene and protein expression was significantly inhibited [266].

Cheng et al. found that graphene/Au composites with a high positive charge could bind to and condense negatively charged siRNA. During nanomaterial synthesis, PEI was used as a reductant and protective reagent, while methoxyl-PEG was used to acquire low cytotoxicity and optimal compatibility. The obtained PEGylated PEI-grafted graphene/Au composites allowed to efficiently transport siRNA into HL-60 cells and downregulate the antiapoptotic protein Bcl-2. Moreover, the nanovector displayed an enhanced photothermal response because of Au nanoparticles under NIR light [267].

In another example, Liu et al. recently developed an amphiphilic and biodegradable copolymer conjugated with folate as the ligand, namely PEI-grafted polycaprolactone-block-poly(ethylene glycol)-folate, which is capable of siRNA delivery through folate-FR recognition. siRNA was effectively delivered into SKOV-3 cells, an FR overexpressing cell line, by the graphene-based nanosystem, and it successfully silenced the expression of the glyceraldehyde 3-phosphate dehydrogenase gene [86].

Li et al. also evaluated a folic acid-modified amphiphilic linear hyperbranched polymer, namely poly(ethylene glycol)-branched polyethyleneimine-poly(ε-caprolactone), to systemically deliver miR-210 into breast cancer cells. This nanosystem linked miR-210 to form a three-layered polyplex through electrostatic interaction. Polyplex was characterized by a high specificity due to cell targeting derived from the recognition of folic acid to overexpressed folate receptor in the cell membrane of breast cancer cells as well as high transfection efficiency in cancer cells. High therapeutic efficacy to inhibit tumor growth in vivo by using a xenograft tumor mouse model was reported [85].

Ewe et al. conducted first study to explore polyethyleneimine-based lipopolyplexes comprising a low-molecular-weight PEI and phospholipids for transporting siRNA. The lipopolyplexes presented in this work are based on cationic polymers such as low-molecular-weight PEI and an already approved liposome. Upon systemic administration in tumor-bearing mice, lipopolyplex-mediated delivery of intact siRNAs was observed. In a PC-3 tumor xenograft model, a considerable inhibition of prostate carcinoma xenograft growth was achieved, paralleled by an ~ 65% survivin knockdown in the tumors [268].

Pourianazar et al. used CpG oligodeoxynucleotide-loaded PAMAM dendrimer-coated magnetic nanoparticles to promote apoptosis in breast cancer cells. CpG oligodeoxynucleotide activate Toll-like receptor 9, which can generate a signal cascade for cell death. The nanosystem was constructed as three-layer magnetic nanoparticles composed of an Fe3O4 magnetic core, an aminosilane interlayer, and a cationic PAMAM dendrimer. CpG oligodeoxynucleotide was attached to the magnetic core due to electrostatic forces. The conducted tests yielded satisfactory toxicity results and proved high apoptotic characteristics of these conjugates for breast cancer cells [269].

Cai et al. reported bioreducible fluorinated peptide dendrimers for efficient and safe siRNA delivery. Reversible crosslinking of fluorinated second generation poly(l-lysine) dendrimers, carrying siVEGF demonstrated excellent VEGF silencing efficacy (~ 65%) and a strong capability for inhibiting HeLa cell proliferation. Furthermore, these polyplexes showed high influence on tumor growth in vitro and in vivo (HeLa tumor xenografts) [270].

Xiong et al. designed a novel supramolecular nanoparticle system with core–shell structure by using cyclodextrin-conjugated poly-l-lysine and HA for co-delivery of gene and chemotherapy agent targeting hepatocellular carcinoma. Taking advantage of conjugation-activated β-cyclodextrin with poly-l-lysine, doxorubicin was included into the hydrophobic cavity of β-cyclodextrin in the constructed complex through host–guest interaction. OligoRNA was further attached in the complexes by electrostatic interaction. Additionally, HA, which is often overexpressed by HCC cells, was coated on the surface of the NPs to construct the HCC-targeted nanoparticle system with classic the so-called “core–shell” structure. The nanoparticles effectively delivered both doxorubicin and oligoRNA into HCC cells through CD44 receptor-mediated endocytosis and significantly inhibited cell proliferation. In the nude mice bearing MHCC-97H tumor, the nanoparticles were efficiently accumulated in the tumor, suggesting their strong hepatoma-targeting capability [271].

Low-molecular-weight polyethyleneimine linked by β-cyclodextrin and conjugated with folic acid nanoparticle was formulated by Zhang et al. to target the delivery of androgen receptor-shRNA to HIPC PC3 (androgen receptor-independent) and 22Rv1 (androgen receptor-dependent) cells lines. Androgen receptor-shRNA-loaded NPs effectively enhanced the radiosensitivity of 22Rv1 cells by selectively knocking down androgen receptor expression, whereas these NPs had no significant effect on PC3 cells and PC3 tumor-bearing mice. Inhibition of cell growth, increased apoptosis, and increased cell cycle arrest in androgen receptor-dependent HIPC in vitro were observed. In vivo androgen receptor-shRNA-decorated NPs could significantly suppress tumor growth and prolong the survival of HIPC tumor-bearing mice. At the molecular level, androgen receptor-shRNA-targeted NPs inhibited DNA damage repair signaling pathways. This study supports the investigation of androgen receptor-shRNA-loaded NPs for the improvement of radiotherapy efficacy in hormone independent prostate cancer [272].

Although bevacizumab, a monoclonal VEGF antibody, was the first FDA-approved antiangiogenic agent for cancer, it use for treatment often led to development of resistance. To overcome these drawbacks, Kim et al. investigated the therapeutic efficacy of siRNA drugs targeting VEGF and the combination of the RNAi drug with bevacizumab. For efficient VEGF-siRNA delivery, chemically polymerized siRNAs were complexed with thiolated-glycol chitosan. The poly-VEGF siRNA and thiolated-glycol chitosan formed stable nanoparticles through electrostatic interaction and chemical crosslinking and showed high accumulation in tumor tissues, resulting in efficient gene silencing. Both VEGF-siRNA nanoparticles and bevacizumab had efficient therapeutic effects on tumor xenograft-bearing Balb/c male nude mice models. It is worthwhile to emphasize that greater therapeutic efficacy was observed when the two distinct VEGF inhibitors were used in combination, thus revealing synergistic effects [273].

Lee et al. presented a dual gene-targeted siRNA and its delivery system to achieve synergistic effects in cancer therapy. Two different sequences of siRNA, VEGF- and Bcl-2-targeting siRNA, were chemically modified and incorporated into glycol chitosan nanoparticles. Dual-poly-siRNA carrying glycol chitosan nanoparticles demonstrated successful dual gene silencing in vitro on PC-3 cells [274].

In the study of Chen et al., VEGF-small interfering RNA was encapsulated into a complex-functionalized magnetic mesoporous silica nanoparticle. The prepared siRNA delivery system readily exhibited cellular internalization and its endosomal escape, resulting in excellent RNAi efficacy without associated cytotoxicity in SKOV3 cells. In in vivo experiments, notable retardation of tumor growth was observed in orthotopic ovarian tumor-bearing mice, which was due to significant inhibition of angiogenesis by systemic administration of this nanocarrier. Furthermore, the described magnetic core nanosystem proved capable of probing the site and size of ovarian cancer in mice on magnetic resonance imaging [275].

Nanopeptide complexes are likely to become highly useful siRNA delivery agents. Bjorge et al. evaluated two different siRNA-binding delivery peptides containing a polyarginine core, and modified by myristoylation and targeting motifs (iRGD or LyP-1). They added a cell-penetrating peptide to assist in endosomal release. The NPs were internalized by the cells, which resulted in knockdown of the corresponding targeted proteins. The peptide with the LyP-1 targeting moiety was more effective at knocking down in MDA-MB-231 breast cancer cells than the peptide with the iRGD motif. Peptide/siRNA complexes simultaneously targeting Stat3 and c-Myc caused a significant reduction of tumor growth [276].

## Conclusion

Nanotechnology promises new engineered materials for medical applications by having new or enhanced physicochemical and biological properties. Over the last years, we have particularly observed a considerable progress in the use of such nanocomposites for cancer nanotechnology. Although some passive targeting agents are still applied for most anticancer therapies in humans, they suffer from numerous limitations such as high administration doses and specific organ toxicities. Therefore, active targeting of drug candidates with nanocomposites used as biomolecule delivery systems is being researched as an alternatives to passive targeting in modern anticancer therapies. There are many nanoplatforms for targeted delivery of biomolecules, such as antibodies, membrane receptors, nucleic acids, aptamers, proteins and peptides, and small entities like folates, vitamins and carbohydrates; however, each of such nano(bio)systems has its own advantages and limitations. It should be noted that the real issue here is to conduct a rigorous investigation of the potential toxicities and risks raised by the use, development, and commercialization of current or new nano-based bioproducts. There is no doubt that the specific properties of nanoscale objects when combined with biomolecules can radically enhance their cellular reactivity. Therefore, there is a great need to understand the complexities and issues of modern toxicology of bioengineered nanocomposites and a need for continued harmonization for risk management. To understand the challenges being faced and the progress already made in cancer nanotechnology, it is worth to further explore next generation bioengineered nanomaterials.

## Abbreviations

BBB: blood–brain barrier
β-CD: β-cyclodextrin
BSA: bovine serum albumin
DOC: docetaxel
DOX: doxorubicin
EGFP: enhanced green fluorescence protein
FR: folate receptor
GO: graphene oxide
GOX: glucose oxidase
Gr: granzyme
HA: hyaluronic acid
HIFU: high intensity focused ultrasound ablation
HUVEC: human umbilical vein endothelial cells
ICAM-1: intercellular adhesion molecule-1
IL10: interleukin 10
MDR: multidrug resistance
MNPs: magnetic nanoparticles
MTX: methotrexate
NIR: near infrared
NPs: nanoparticles
PEG: polyethylene glycol
PEI: polyethyleneimine
PET: positron emission tomography
PLGA: poly(lactic-*co*-glycolic acid)
Pt(IV): cisplatin
PVP: polyvinylpyrrolidone
rGO: reduced graphene oxide
ROS: reactive oxygen species
SOD: superoxide dismutase
Tf: transferrin
TNF: tumor necrosis factor
VEGF: vascular endothelial growth factor
